# The Malaria Parasite's Lactate Transporter PfFNT Is the Target of Antiplasmodial Compounds Identified in Whole Cell Phenotypic Screens

**DOI:** 10.1371/journal.ppat.1006180

**Published:** 2017-02-08

**Authors:** Sanduni V. Hapuarachchi, Simon A. Cobbold, Sarah H. Shafik, Adelaide S. M. Dennis, Malcolm J. McConville, Rowena E. Martin, Kiaran Kirk, Adele M. Lehane

**Affiliations:** 1 Research School of Biology, Australian National University, Canberra, ACT, Australia; 2 Department of Biochemistry and Molecular Biology, Bio21 Molecular Science and Biotechnology Institute, University of Melbourne, Melbourne, VIC, Australia; U Tex SouthWestern, UNITED STATES

## Abstract

In this study the ‘Malaria Box’ chemical library comprising 400 compounds with antiplasmodial activity was screened for compounds that perturb the internal pH of the malaria parasite, *Plasmodium falciparum*. Fifteen compounds induced an acidification of the parasite cytosol. Two of these did so by inhibiting the parasite’s formate nitrite transporter (PfFNT), which mediates the H^+^-coupled efflux from the parasite of lactate generated by glycolysis. Both compounds were shown to inhibit lactate transport across the parasite plasma membrane, and the transport of lactate by PfFNT expressed in *Xenopus laevis* oocytes. PfFNT inhibition caused accumulation of lactate in parasitised erythrocytes, and swelling of both the parasite and parasitised erythrocyte. Long-term exposure of parasites to one of the inhibitors gave rise to resistant parasites with a mutant form of PfFNT that showed reduced inhibitor sensitivity. This study provides the first evidence that PfFNT is a druggable antimalarial target.

## Author Summary

The emergence and spread of *Plasmodium falciparum* strains resistant to leading antimalarial drugs has intensified the need to discover and develop drugs that kill the parasite via new mechanisms. Here we screened compounds that are known to inhibit *P*. *falciparum* growth for their effects on the pH inside the parasite. We identified fifteen compounds that decrease the pH inside the parasite, and determined the mechanism by which two of these, MMV007839 and MMV000972, disrupt pH and kill the parasite. The two compounds were found to inhibit the *P*. *falciparum* formate nitrite transporter (PfFNT), a transport protein that is located on the parasite surface and that serves to remove the waste product lactic acid from the parasite. The compounds inhibited both the H^+^-coupled transport of lactate across the parasite plasma membrane and the transport of lactate by PfFNT expressed in *Xenopus* oocytes. In addition to disrupting pH, PfFNT inhibition led to a build-up of lactate in the parasite-infected red blood cell and the swelling of both the parasite and the infected red blood cell. Exposing parasites to MMV007839 over a prolonged time period gave rise to resistant parasites with a mutant form of PfFNT that was less sensitive to the compound. This study validates PfFNT as a novel antimalarial drug target.

## Introduction

The most virulent malaria parasite, *Plasmodium falciparum*, was responsible for the majority of the 214 million malaria cases and 438,000 malaria-attributable deaths estimated to have occurred in 2015 [1]. The parasite has developed resistance to most of the drugs deployed against it [2], and it is imperative that new drugs be developed to protect or replace existing therapies. Significant progress has been made in recent years in developing new antimalarials [3]; however, many of the compounds under development are structurally related to previous or current antimalarial drugs and there is an urgent need to identify novel lead-drug compounds that act on hitherto unexploited parasite targets and create improved options for resistance-deterring combination therapies. Such therapies should, ideally, contain at least two drugs that act on separate targets [4].

Recent large-scale whole cell phenotypic screens have uncovered tens of thousands of novel inhibitors of the *in vitro* growth of asexual *P*. *falciparum* parasites in human erythrocytes [5–7]. In a bid to further research into the novel antiplasmodial chemotypes, the Medicines for Malaria Venture (MMV) compiled the open access ‘Malaria Box’, a collection of 400 structurally-diverse compounds selected from the drug screen hits for which the mechanisms of action were not known [8]. Determining the mechanisms of action of these antiplasmodial compounds has the potential to uncover aspects of *P*. *falciparum* biology that can be exploited as drug targets.

The mechanism by which *P*. *falciparum* generates ATP presents potential vulnerabilities. In particular, the (disease-causing) asexual intraerythrocytic stages of *P*. *falciparum* rely primarily on glycolysis for energy metabolism [9]. Human erythrocytes infected with trophozoite-stage parasites devour glucose up to 100 times faster than uninfected erythrocytes [10], and generate large quantities of lactate, which is exported via a lactate:H^+^ symport mechanism [11]. Both glucose uptake and lactate export are likely to be essential for maintaining the energy requirements, intracellular pH and osmotic stability of these parasite stages. The *P*. *falciparum* hexose transporter (PfHT) mediates the uptake of glucose into the parasite and has been of interest as a drug target for some time [12, 13]. Recently, a compound from the Malaria Box was found to inhibit PfHT potently (with a 50% inhibitory concentration (IC_50_) of ~ 50 nM) while also displaying a high degree of selectivity for PfHT over the human glucose transporter GLUT1 [14].

A transport protein that mediates the efflux of lactate from the intraerythrocytic malaria parasite has recently been identified and characterised [15–17]. The protein belongs to the microbial formate nitrite transporter (FNT) family, localises to the parasite plasma membrane (as well as to the membrane bounding the parasite’s internal digestive vacuole), and transports lactate, as well as a variety of other monocarboxylates, in a pH-dependent manner that is consistent with H^+^-coupled transport [16, 17]. The FNT family is structurally unrelated to the monocarboxylate transporters that export lactate from human cells [16, 17].

In this study we have screened the Malaria Box for compounds that alter the intracellular pH of asexual trophozoite-stage parasites. Fifteen of the 400 compounds were found to acidify the parasite cytosol. Of these, two compounds were found to exert their effects on the parasite’s cytosolic pH by targeting the H^+^-coupled efflux of lactate via PfFNT.

## Results

### Screening the Malaria Box compounds for effects on parasite pH

In a previous study we showed that 28 compounds from the Malaria Box, when added to isolated asexual *P*. *falciparum* parasites, give rise to a cytosolic alkalinisation, together with an increase in the cytosolic [Na^+^] [18]. The data are consistent with the compounds inhibiting the putative Na^+^/H^+^ ATPase PfATP4 [19]. Here, we screened the remaining 372 Malaria Box compounds for their effects, at 1 μM, on parasite cytosolic pH. The screen entailed adding a 1 μL aliquot of a 1 mM stock of each compound to a 1 mL suspension of trophozoite-stage *P*. *falciparum* parasites that had been isolated from their host erythrocytes by brief exposure to saponin and loaded with the pH-sensitive fluorescent dye BCECF.

**Fig 1:**
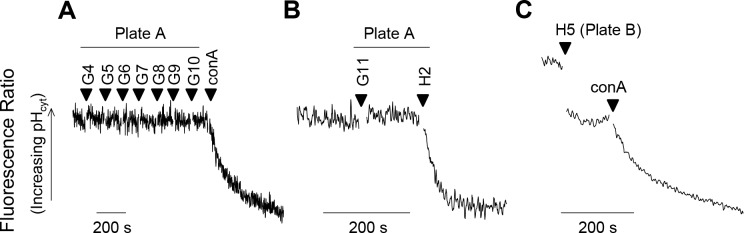
Representative traces illustrating the strategy employed to screen the Malaria Box for compounds that perturb the cytosolic pH (pH_cyt_) of the parasite. Malaria Box compounds (1 μM; from the May 2012 Malaria Box batch; identified here by their positions on the plates) were added successively to BCECF-loaded isolated 3D7 *P*. *falciparum* trophozoites (suspended at 37°C in pH 7.10 Experimental Saline Solution). A maximum of nine inactive compounds were tested on a single batch of parasites before the V-type H^+^-ATPase inhibitor concanamycin A (conA; 100 nM) was added as a positive control to ensure that a pH change was still detectable (A). A batch of parasites was also replaced with a new batch after a hit was detected (B) or a compound caused an optical effect (seen as an instantaneous perturbation of the fluorescence signal; C). In the latter case concanamycin A (100 nM) was added before changing to another batch of parasites. The purpose of adding the concanamycin A was to confirm (or otherwise) that any such optical effect did not obscure the pH-responsiveness of the fluorescence signal.

The fluorescence of the parasite suspension was monitored continuously. If, on addition of a compound, no change in fluorescence was observed after 2 min, another compound was added to the same cell suspension. A maximum of 9 compounds were added successively to a single batch of cells before concanamycin A (100 nM), an inhibitor of the parasite’s H^+^-extruding V-type ATPase that has been shown previously to acidify the cytosol in *P*. *falciparum* [20], was added as a positive control to confirm that a pH change was still detectable in those cells (Fig 1A).

Fifteen of the 372 Malaria Box compounds tested were found to give rise to an immediate-onset gradual decrease in fluorescence ratio (i.e. a decrease in the ratio of the fluorescence measured using two excitation wavelengths: 495 nm [pH-sensitive numerator] and 440 nm [pH-insensitive denominator]). In each case the decrease in fluorescence ratio resulted from a decrease in the pH-sensitive numerator, consistent with the cells having undergone cytosolic acidification (Fig 1B). The 15 compounds that induced an acidification in the initial screen (and for which structures are shown in S1 Table) were all re-tested in at least two further pH experiments and their effects on the cytosolic pH of the parasite confirmed.

The 15 compounds were also tested for their effects (at 1 μM and 5 μM) on the pH of the parasite’s digestive vacuole. This organelle is acidic, with an estimated pH value of approximately 5 [21–23]. Seven of the 15 compounds dissipated the pH gradient across the digestive vacuole membrane (causing its alkalinisation; S1 Table; S1 Fig).

Of the 372 Malaria Box compounds tested for their effects on cytosolic pH, 23 gave rise to effects on the fluorescence ratio that were considered likely to be ‘optical effects’ rather than genuine pH changes. Such effects arise from either an intrinsic fluorescence of the compound or an interaction between the compound and the fluorescent dye. In each case the change of the fluorescence ratio was abrupt rather than gradual (see example in Fig 1C). Furthermore, in most (20 out of 23) cases the change in fluorescence ratio resulted in full or in part from a change in the nominally pH-*insensitive* fluorescence emanating from excitation at 440 nm (the denominator). After an optical effect was encountered, concanamycin A (100 nM) was added to the cells to determine whether a pH change was still detectable (which, in each case, it was, ruling out the possibility that the compound that caused the optical effect had also dissipated the pH gradient across the plasma membrane), and the cells were replaced with a new batch of cells before screening further Malaria Box compounds.

### The effects of MMV007839 and MMV000972 on parasite pH are consistent with inhibition of lactate transport

One mechanism by which a compound might induce a cytosolic acidification is through inhibition of one (or more) of the plasma membrane proteins that mediate the efflux of H^+^ from the parasite. Lactate, produced in large quantities by the parasite as the end-product of glycolysis, is excreted via a coupled lactate:H^+^ transporter [11, 16, 17], raising the possibility that this protein might be a candidate target for some of the compounds identified as hits in the initial screen. Compounds that inhibit the lactate:H^+^ transporter might be expected to: (i) have no effect on cytosolic pH in parasites suspended in a glucose-free saline, as under this condition glycolysis is not operating, and there is therefore no production of lactate; (ii) prevent the lactate:H^+^ transporter-mediated cytosolic acidification that results from the exposure of a parasite to a large *inward* lactate gradient. Although the physiological role of the transporter is to mediate the net export of lactate, down the normally-outward lactate concentration gradient, the transporter is bidirectional and, under conditions of an imposed inward lactate concentration gradient (as results from the addition of a high concentration of lactate to the extracellular medium) it mediates a net influx of lactate:H^+^, resulting in a cytosolic acidification [11].

**Fig 2:**
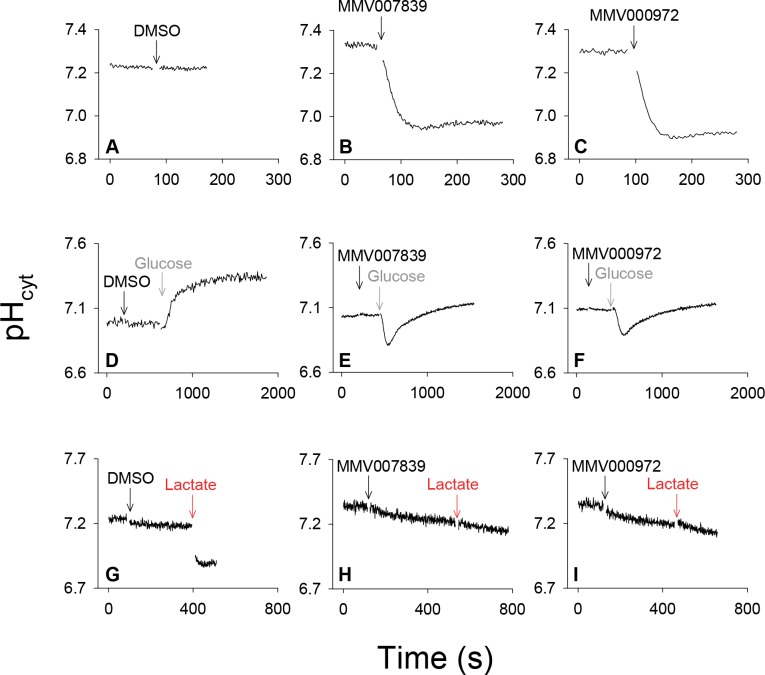
Fluorescence traces showing the effects of DMSO (solvent control), MMV007839 and MMV000972 on the cytosolic pH (pH_cyt_) of 3D7 *P*. *falciparum* trophozoites under varying conditions. Each trace is representative of those obtained in at least three independent experiments. (A-C) The effects of DMSO (0.1% v/v; solvent control; A), MMV007839 (1 μM; B) and MMV000972 (1 μM; C) on the cytosolic pH of parasites suspended in (glucose-containing) Experimental Saline Solution (pH 7.10) at 37°C. (D-F) The effects of DMSO (0.25% v/v; solvent control; D), MMV007839 (2.5 μM; E), and MMV000972 (2.5 μM; F) on the cytosolic pH of parasites suspended (for 10 min prior to commencing recording, and throughout the initial recording period) in glucose-free Experimental Saline Solution (pH 7.10, 37°C), and the effects of the subsequent addition of glucose (22 mM) to the parasite suspension. (G-I) The effects of adding DMSO (0.25% v/v; solvent control; G), MMV007839 (2.5 μM; H), and MMV000972 (2.5 μM; I), followed by sodium L-lactate (10 mM), on the cytosolic pH of parasites suspended at 4°C in Experimental Saline Solution (pH 7.10).

When tested at concentrations of 2.5–5 μM, only two of the 15 pH-lowering hits, MMV007839 and MMV000972 (which are structurally similar to one another; S1 Table) demonstrated both features that would be expected from a complete inhibition of lactate:H^+^ transport (Fig 2). The addition of MMV007839 or MMV000972 to isolated parasites suspended in a glucose-containing medium (in which glycolysis was active) resulted in cytosolic acidification (Fig 2B and 2C). The exposure of parasites to the solvent alone (0.1% v/v DMSO) had no effect on pH (Fig 2A). When isolated parasites are suspended in glucose-free medium, glycolysis ceases, the intracellular ATP concentration decreases to zero, and the parasite is unable to maintain the activity of the plasma membrane V-type H^+^-ATPase which serves to generate and maintain an inward H^+^ electrochemical gradient [24, 25]. As a result, the resting pH decreases to 7.0–7.1 (i.e. close to the extracellular pH) compared with 7.2–7.3 in glucose-replete cells. On addition of MMV007839 or MMV000972 to isolated parasites suspended in glucose-free medium (in which lactate production is eliminated) there was no change in the resting pH (Fig 2E and 2F), consistent with the acidification seen in glucose-replete cells being due to the inhibition of lactate efflux.

On restoration of glucose to parasites suspended in glucose-free medium (in the absence of inhibitors) pH increased as ATP levels, and hence the activity of the H^+^-extruding V-type H^+^-ATPase, were restored (Fig 2D). When the same manoeuvre was applied to parasites exposed to either MMV007839 or MMV000972, there was an initial acidification (consistent with lactate production having been restored and with the compounds inhibiting the efflux of the newly-generated lactate:H^+^; Fig 2E and 2F). However, after ~ 2 min the cells underwent a gradual alkalinisation, restoring the cytosolic pH to approximately 7.1 (Fig 2E and 2F). The basis for this recovery is not understood; no such recovery was observed when the compounds were added to parasites maintained continuously in the presence of glucose (Fig 2B and 2C).

MMV007839 and MMV000972 were then tested for their ability to inhibit the lactate:H^+^ transporter-mediated acidification of the parasite cytosol that normally occurs when lactate is added to the extracellular medium. This was tested at 4°C (Fig 2G–2I) to reduce the ability of the H^+^-extruding V-type H^+^-ATPase to counteract the lactate-mediated pH change [11]. At 4°C the acidification of the parasite cytosol induced by the addition of MMV007839 and MMV000972 was less pronounced than that seen at 37°C (cf. Fig 2B and 2C and Fig 2H and 2I), reflecting the reduced rate of glycolysis and hence reduced rate of lactate production at the lower temperature. The addition of 10 mM L-lactate to parasites (following a pre-treatment with solvent (0.25% v/v DMSO), which had no effect on cytosolic pH) induced an abrupt acidification of the parasite cytosol (Fig 2G), consistent with lactate entering the parasite rapidly in symport with H^+^. On addition of 10 mM L-lactate to parasites that had been pre-treated with MMV007839 or MMV000972, there was no such abrupt acidification (Fig 2H and 2I). Rather, the slow acidification mediated by the compounds continued at the same rate as that seen prior to the addition of L-lactate. For MMV007839 the rates of acidification before and after the addition of lactate were 0.011 ± 0.003 pH unit/min (mean ± SEM) and 0.011 ± 0.004 pH unit/min (n = 3; *P* = 0.9; paired t-test). For MMV000972 the rates were 0.012 ± 0.003 pH unit/min and 0.015 ± 0.006 pH unit/min (n = 3; *P* = 0.5). These data are consistent with the two MMV compounds inhibiting the lactate:H^+^ transporter and, thereby, the influx of lactate:H^+^ into the parasite.

### *In vitro* generated MMV007839-resistant parasites have a mutation in PfFNT

The pH data obtained with the isolated parasites provide indirect evidence that MMV007839 and MMV000972 inhibit lactate:H^+^ symport across the parasite plasma membrane but do not reveal the molecular identity of the target. To gain information on the possible molecular target of the compounds, we cultured a recently-cloned *P*. *falciparum* Dd2 parasite line in the presence of increasing concentrations of MMV007839 until parasites showing resistance to the growth-inhibiting effect of this compound emerged. Two independent cultures (referred to here as ‘MMV007839-selected cultures A and B’) were initially maintained in the presence of 100 nM MMV007839, and the concentration subsequently increased incrementally over the course of six weeks.

The parasites from both cultures were tested, along with the Dd2 parental line, for their sensitivity to growth inhibition by MMV007839 and MMV000972. Dd2 parental parasites had an IC_50_ value for MMV007839 of 0.14 ± 0.02 μM and an IC_50_ value for MMV000972 of 1.8 ± 0.2 μM. Parasites from both MMV007839-selected cultures were highly resistant to both compounds, with IC_50_ values > 280-fold higher than that of the Dd2 parent for MMV007839, and > 30-fold higher than that of the Dd2 parent for MMV000972 (*P* < 0.001; paired t-tests; Table 1). The responses of parasites in the MMV007839-selected cultures to chloroquine and artemisinin were not significantly different from those of the Dd2 parental parasites (*P* > 0.1; Table 1).

**Table 1:** PfFNT mutation and growth inhibition by various compounds in parasites generated by exposure to increasing concentrations of MMV007839.

	Mutation in PfFNT (relative to Dd2 parent)	IC_50_ value (μM)		IC_50_ value (nM)	
		MMV007839	MMV000972	Chloroquine	Artemisinin
Dd2 parent	-	0.14 ± 0.02 (7)	1.8 ± 0.2 (3)	149 ± 6 (7)	15.6 ± 0.0 (3)
MMV007839-selected culture A	Gly107Ser	35.8 ± 2.7*** (7)	59.6 ± 0.4*** (3)	151 ± 7^ns^ (7)	22.0 ± 3.3^ns^ (3)
MMV007839-selected culture B	Gly107Ser	37.5 ± 2.7*** (6)	59.7 ± 1.4*** (3)	147 ± 5^ns^ (6)	16.5 ± 0.4^ns^ (3)
3D7 (our laboratory)	ND	0.26 ± 0.03 (5)	ND	ND	ND

The data are the mean ± SEM from the number of independent experiments indicated in brackets. ND, not determined. The IC_50_ values obtained for MMV007839-selected cultures A and B were compared to those of the Dd2 parent using paired t-tests; ns, not significant

***P < 0.001.

Genomic DNA was extracted from both of the MMV007839-selected cultures as well as from the parental Dd2 culture. The entire *PfFNT* gene was then amplified by PCR and the coding regions sequenced. Parasites from each of the MMV007839-selected cultures were found to have a mutation that was not present in the Dd2 parental parasites or in the 3D7 reference sequence. In both MMV007839-selected cultures (A and B), the mutation in the coding sequence was G319A, which introduces a Gly107Ser mutation into the PfFNT protein. The finding of a mutation in the lactate:H^+^ transporter PfFNT in the MMV007839-selected cultures is consistent with PfFNT inhibition being the mechanism of action of MMV007839 and MMV000972. The parental and MMV007839-selected Dd2 parasites also had two polymorphisms in the *PfFNT* coding sequence relative to the 3D7 strain (PlasmoDB ID PF3D7_0316600): a C475G change, which codes for a His159Asp mutation, and a A756G change (synonymous).

### MMV007839 and MMV000972 inhibit L-[^14^C]lactate transport by *P*. *falciparum* parasites, with their potency reduced in parasites expressing PfFNT^Gly107Ser^

The effects of MMV007839 and MMV000972 on lactate transport across the parasite plasma membrane were investigated by measuring the uptake of L-[^14^C]lactate by saponin-isolated trophozoite-stage parasites in the presence and absence of the compounds. As noted above, although the physiological role of the transporter is to facilitate the net export of lactate from the parasite, the transporter is bidirectional, and measuring the unidirectional flux of L-[^14^C]lactate *into* the parasite provides a convenient means of monitoring the activity of the transporter. It has been shown previously that the uptake of external L-[^14^C]lactate by isolated parasites is enhanced at lower pH values, consistent with the transporter being H^+^-coupled [11]. The L-[^14^C]lactate uptake experiments were therefore performed on isolated parasites suspended in an acidic (pH 6.1) medium, at 4°C (to slow the transport process). The level of intracellular L-[^14^C]lactate accumulation was determined 20 s after the addition of the radiolabel to parasites. Under these conditions (i.e. pH 6.1, 4°C) the 20 s incubation falls within the initial linear phase of uptake of the radiolabel and the measured uptake therefore reflects the lactate influx rate (our own preliminary experiments and [11]).

In isolated 3D7 parasites to which had been added DMSO (0.4% v/v, as a solvent control) the L-[^14^C]lactate ‘distribution ratio’ (i.e. the intracellular L-[^14^C]lactate concentration divided by the extracellular L-[^14^C]lactate concentration) at 20 s reached a value of 6.7 ± 0.4 (mean ± SEM; n = 4; S2 Fig). As has been reported previously [16], the broad-specificity anion transport inhibitor NPPB (50 μM) slowed L-[^14^C]lactate influx, reducing the distribution ratio at 20 s to 0.4 ± 0.1 (*P* < 0.001; one-way ANOVA with *post hoc* Tukey test; S2 Fig). MMV007839 (0.5 μM) inhibited L-[^14^C]lactate influx to a similar degree (*P* < 0.001; S2 Fig), with 2 μM MMV007839, as well as 0.5 μM and 2 μM MMV000972, causing complete inhibition of L-[^14^C]lactate uptake (*P* < 0.001; S2 Fig).

**Fig 3:**
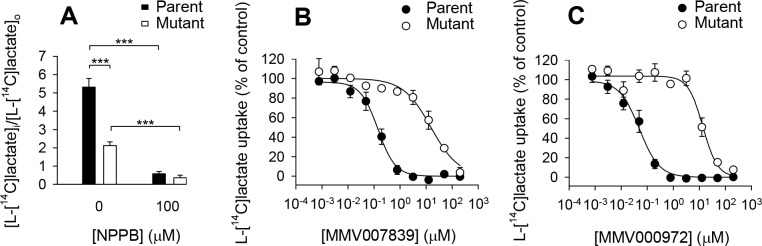
L-[^14^C]lactate influx into PfFNT^Gly107Ser^ mutant parasites and Dd2 parental parasites. (A) The uptake of L-[^14^C]lactate by isolated trophozoite-stage PfFNT^Gly107Ser^ mutant parasites (white bars) and Dd2 parental parasites (black bars) in the absence of compound (0.4% v/v DMSO; solvent control) and in the presence of the non-specific anion transport inhibitor NPPB (100 μM). The mean distribution ratio (i.e., [intracellular L-[^14^C]lactate]/[extracellular L-[^14^C]lactate]) and SEM from nine (parent) or seven (mutant) independent experiments for the control, and from three independent experiments for NPPB, are shown. The results of unpaired t-tests are shown; ***P < 0.001. (B-C) The effects of MMV007839 (B) and MMV000972 (C) on L-[^14^C]lactate uptake into isolated trophozoite-stage PfFNT^Gly107Ser^ mutant parasites (white symbols) and Dd2 parental parasites (black symbols). L-[^14^C]lactate uptake in the presence of each concentration of MMV compound is expressed as a percentage of that observed when there was no inhibition of transport. The data shown are the average ± SEM from *n* independent experiments performed on different days, where *n* was seven for the MMV007839 data with parental parasites, three for the MMV007839 data with PfFNT^Gly107Ser^ mutant parasites, three for the MMV000972 data with parental parasites, and four for the MMV000972 data with PfFNT^Gly107Ser^ mutant parasites (with the exception of the lowest concentration, for which there was one less experiment in all cases). In all cases (panels A-C), the experiments were performed at 4°C in pH 6.1 Experimental Saline Solution, and parasites were preincubated with the compound (at the concentration indicated) or DMSO (0.4% v/v; solvent control) for 1 min prior to the addition of L-[^14^C]lactate (1.3 μM). The concentrations of compound and DMSO after the addition of L-[^14^C]lactate were the same as those present during the preincubation. The uptake of L-[^14^C]lactate was measured over 20 s.

A comparison of the uptake of L-[^14^C]lactate into the PfFNT^Gly107Ser^ mutant parasites (from MMV007839-selected culture B) and their parental parasites revealed that transport was slowed significantly in the mutant strains (*P* < 0.001; unpaired t-test; Fig 3A). In the PfFNT^Gly107Ser^ mutant parasites the L-[^14^C]lactate distribution ratio, as measured at 20 s, reached a value of 2.1 ± 0.2 (mean ± SEM; n = 7), compared to a value of 5.3 ± 0.5 (n = 9) in the parental parasites. This finding suggests that the Gly107Ser mutation in PfFNT causes some impairment of its function. NPPB (100 μM) reduced the rate of L-[^14^C]lactate influx into both PfFNT^Gly107Ser^ mutant and parental parasites (Fig 3A; *P* < 0.001; unpaired t-tests).

The concentration dependence of the inhibition of L-lactate influx by the two MMV compounds in the PfFNT^Gly107Ser^ mutant parasites (MMV007839-selected culture B) was compared to that in the parental line. Both compounds were potent inhibitors of L-[^14^C]lactate uptake by Dd2 (parental) parasites (Fig 3B and 3C). The IC_50_ values for MMV007839 and MMV000972 in these experiments were 158 ± 42 nM (mean ± SEM; n = 7) and 49 ± 14 nM (n = 3), respectively. In the PfFNT^Gly107Ser^ mutant parasites the efficacy of MMV007839 and MMV000972 at inhibiting L-[^14^C]lactate transport was significantly reduced (Fig 3B and 3C; *P* ≤ 0.001; unpaired t-tests) with IC_50_ values of 16.3 ± 4.4 μM (n = 3) and 15.2 ± 1.9 μM (n = 4), respectively.

The finding of a reduced rate of L-[^14^C]lactate transport across the plasma membrane of PfFNT^Gly107Ser^ mutant parasites compared to parental parasites (Fig 3A) raised the question of whether the Gly107Ser mutation in PfFNT might be associated with a reduction in parasite fitness. To investigate this, we compared the growth rates of two PfFNT^Gly107Ser^ mutant parasite clones (generated from MMV007839-selected culture B by limiting dilution) with that of the parental Dd2 clone. We performed competition experiments in which mutant and parental parasites were mixed in an approximately 1:1 ratio, and their relative proportions monitored over time. These experiments revealed that the proportion of mutant and parental parasites remained approximately constant over the course of three weeks (S3 Fig). Thus, the Gly107Ser mutation in PfFNT was not associated with a decrease in the growth rate of asexual blood-stage parasites, suggesting that the parasite can withstand a substantial reduction in the rate of PfFNT-mediated lactate transport before its viability is compromised.

### MMV007839 and MMV000972 inhibit L-[^14^C]lactate transport by PfFNT expressed in *Xenopus* oocytes, with PfFNT^Gly107Ser^ being less sensitive to inhibition than native PfFNT

To determine directly whether PfFNT is inhibited by MMV007839 and MMV000972, and if so whether the Gly107Ser mutation reduces the sensitivity of PfFNT to inhibition by the compounds, we expressed native PfFNT and PfFNT^Gly107Ser^ in *Xenopus laevis* oocytes.

**Fig 4:**
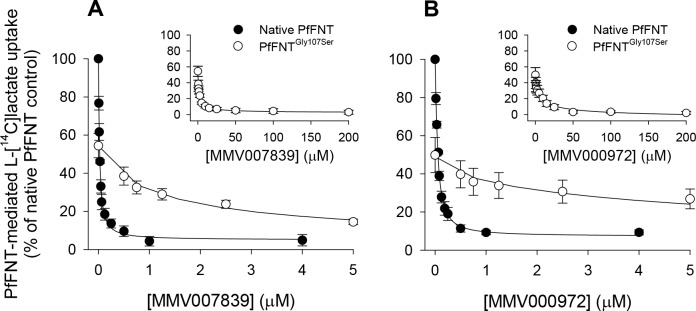
The effects of MMV007839 and MMV000972 on PfFNT-mediated L-[^14^C]lactate uptake by *Xenopus* oocytes expressing native PfFNT or PfFNT^Gly107Ser^. The PfFNT-mediated uptake of L-[^14^C]lactate by oocytes expressing native PfFNT (black symbols) or oocytes expressing PfFNT^Gly107Ser^ (white symbols) was measured in the presence of a range of different concentrations of MMV007839 (A) and MMV000972 (B). The component of transport attributable to PfFNT (or to PfFNT^Gly107Ser^) was determined by subtracting the uptake of L-[^14^C]lactate into non-injected oocytes (averaged from the values obtained across the full range of inhibitor concentrations tested, noting that the uptake of L-[^14^C]lactate into non-injected oocytes was unaffected by either inhibitor; S4 Fig) from the uptake of L-[^14^C]lactate measured (at each inhibitor concentration) into oocytes expressing either native PfFNT or PfFNT^Gly107Ser^. In all panels, the PfFNT-mediated uptake of L-[^14^C]lactate is expressed as a percentage of that determined for native PfFNT in the absence of MMV compound (0.4% v/v DMSO; solvent control). To enable a visual comparison between the native PfFNT and PfFNT^Gly107Ser^ datasets, only part of the concentration range used for the latter is shown in the main panels. The insets show the data obtained for PfFNT^Gly107Ser^ over the full concentration range tested. In all cases, the data are averaged from four independent experiments (using oocytes from different frogs) and are shown ± SEM. The uptake was measured at 27.5°C over 10 min at pH 6.4 using 2.7 μM L-[^14^C]lactate and 600 μM unlabelled L-lactate.

Consistent with our observations in parasites (Fig 3A), which revealed that the rate of L-[^14^C]lactate transport into PfFNT^Gly107Ser^ mutant parasites was only 39 ± 5% (mean ± SEM; n = 7) of that observed in the parental parasites, *Xenopus* oocytes expressing the mutant PfFNT^Gly107Ser^ protein were found to be significantly impaired in their ability to transport L-[^14^C]lactate relative to oocytes expressing native PfFNT (Fig 4). In the absence of MMV007839 or MMV000972, the PfFNT-mediated component of L-[^14^C]lactate transport into oocytes expressing PfFNT^Gly107Ser^ was only 52 ± 7% (mean ± SEM; n = 4) of that measured in oocytes expressing native PfFNT (*P* < 0.001; one-way ANOVA with *post hoc* Tukey test).

MMV007839 and MMV000972 inhibited L-[^14^C]lactate uptake via both the native PfFNT and PfFNT^Gly107Ser^ transporters (Fig 4, S4 Fig), whilst causing no changes in L-[^14^C]lactate transport into the control (non-injected) oocytes (S4 Fig). Much higher concentrations of the compounds were required to inhibit L-[^14^C]lactate transport via PfFNT^Gly107Ser^ compared to those found to inhibit transport via native PfFNT (Fig 4). The IC_50_ values for the inhibition of PfFNT-mediated L-[^14^C]lactate transport by MMV007839 were 22.5 ± 1.4 nM (mean ± SEM; n = 4) for native PfFNT and 1.3 ± 0.2 μM for PfFNT^Gly107Ser^ (*P* < 0.001; one-way ANOVA with *post hoc* Tukey test). For MMV000972, the IC_50_ values for native PfFNT and PfFNT^Gly107Ser^ were 50 ± 1 nM and 6.3 ± 0.9 μM, respectively (*P* < 0.001). These results provide direct evidence that PfFNT is inhibited by MMV007839 and MMV000972 and that the Gly107Ser mutation greatly reduces the sensitivity of PfFNT to inhibition by these compounds.

The specificity of the inhibition by the MMV compounds of the transport of L-[^14^C]lactate via PfFNT was investigated by testing the effects of the compounds on [^3^H]chloroquine transport by oocytes expressing a chloroquine-transporting isoform of the *P*. *falciparum* chloroquine resistance transporter [26] (PfCRT; from the chloroquine-resistant Dd2 strain) and on [^3^H]hypoxanthine transport by oocytes expressing the *P*. *falciparum* equilibrative nucleoside/nucleobase transporter PfENT1 [27, 28]. Neither compound had a statistically significant effect on the transport of [^3^H]chloroquine via PfCRT (S5 Fig). Neither compound inhibited [^3^H]hypoxanthine uptake by PfENT1-expressing oocytes (S5 Fig), although 1 μM MMV000972 caused a slight increase in [^3^H]hypoxanthine uptake by PfENT1-expressing oocytes (*P* < 0.05; one-way ANOVA with *post hoc* Tukey test).

### Inhibition of PfFNT leads to a build-up of lactate and swelling of the parasite and infected erythrocyte

The effect of MMV007839 (the more potent of the two MMV compounds at inhibiting parasite growth) on the metabolite profile of erythrocytes infected with mature 3D7 trophozoites was assessed using a previously-described untargeted LC-MS approach [29]. The compound was added at approximately 20× IC_50_ (Table 1; 6 μM) and samples were taken for metabolite profiling at 1 h, 3 h and 6 h after the addition. Exposure of 3D7 parasites to 6 μM MMV007839 for 1 h or 3 h did not significantly affect their viability (*P* > 0.14; paired t-tests; S6 Fig), assessed by exposing parasitised erythrocytes to the inhibitor for the specified time, then removing the inhibitor and culturing the parasites for a further three days and measuring the final parasitaemia. By contrast, exposure for 6 h resulted in a reduction in parasitaemia (as measured three days after the exposure) to 49 ± 7% (mean ± SEM; n = 3) of control levels (*P* = 0.007; S6 Fig).

**Fig 5:**
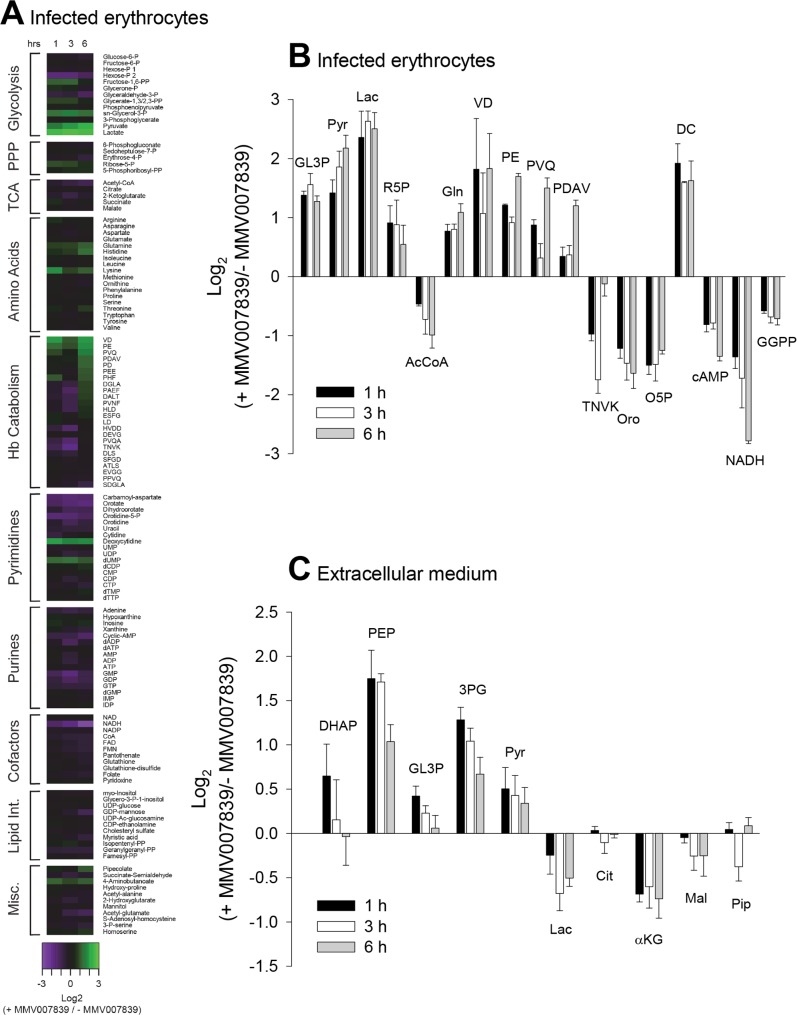
The effect of MMV007839 on the metabolite profile of parasitised erythrocytes. (A) Relative amounts of intracellular metabolites in MMV007839-treated parasitised erythrocytes (infected with the 3D7 strain) and untreated parasitised erythrocytes. The data shown are the mean log_2_ ratios of the level of each metabolite in the MMV007839-treated samples relative to its level in the untreated samples (+ MMV007839/- MMV007839), determined 1 h, 3 h and 6 h after the addition of MMV007839, obtained from three independent experiments. The concentration of MMV007839 used (6 μM) corresponds to approximately 20× the IC_50_ value for growth inhibition of 3D7 parasites. (B) The log_2_ ratios of metabolite levels in MMV007839-treated samples relative to those in untreated samples for all metabolites of known identity for which a statistically significant difference in abundance was observed between MMV007839-treated and untreated infected erythrocytes (see S1 Data for statistical analysis). The data shown are the mean + SEM from three independent experiments. GL3P, sn-glycerol-3-phosphate; Pyr, pyruvate; Lac, lactate; R5P, ribose-5-phosphate; AcCoA, acetyl coenzyme A; Gln, glutamine; VD, Val-Asp; PE, Pro-Glu; PVQ, Pro-Val-Gln; PDAV, Pro-Asp-Ala-Val; TNVK, Thr-Asn-Val-Lys; Oro, orotate; O5P, orotidine-5-phosphate; DC, deoxycytidine; GGPP, geranylgeranyl-pyrophosphate. (C) Relative amounts of metabolites in the extracellular medium in suspensions containing MMV007839-treated and untreated parasitised erythrocytes. The data shown are the mean log_2_ ratios (+ MMV007839/- MMV007839) + SEM from three independent experiments. There was a statistically significant difference in the concentrations of 3-phosphoglycerate and phosphoenolpyruvate in the extracellular medium between MMV007839-treated and untreated cell suspensions (see S1 Data). DHAP, glycerone phosphate; PEP, phosphoenolpyruvate; 3PG, 3-phosphoglycerate; Cit, citrate; αKG, 2-ketoglutarate; Mal, malate; Pip, pipecolate.

The levels of a number of metabolites within infected erythrocytes were found to be affected by MMV007839 (Fig 5; S1 Data). For those metabolites for which differences in abundance between MMV007839-treated and control samples were statistically significant, the relative levels (i.e. the level of the metabolite in MMV007839-treated cells relative to that in untreated cells) are shown in Fig 5B. Lactate was the metabolite that showed the greatest increase in abundance in MMV007839-treated infected erythrocytes relative to untreated infected erythrocytes (a 6-fold increase at each time point; Fig 5A and 5B; S1 Data), consistent with the MMV compound inhibiting the efflux of lactate from the parasite. There was also a significant elevation of pyruvate (the metabolite directly upstream of lactate), the glycolytic metabolite sn-glycerol-3-phosphate, and a number of peptides, in MMV007839-exposed cells (Fig 5A and 5B).

The levels of a number of metabolites were decreased in MMV007839-treated infected erythrocytes relative to untreated infected erythrocytes. These included NADH and the pyrimidine precursors orotate and orotidine-5-phosphate (Fig 5A and 5B).

The levels of a range of metabolites in the extracellular medium were also analysed (Fig 5C). Extracellular lactate and α-ketoglutarate levels were decreased in MMV007839-treated samples relative to untreated samples, whereas the levels of pyruvate, phosphoenolpyruvate and 3-phosphoglycerate were increased. Phosphoenolpyruvate and 3-phosphoglycerate were the only metabolites for which differences in abundance between MMV007839-treated and untreated samples were statistically significant (S1 Data).

**Fig 6:**
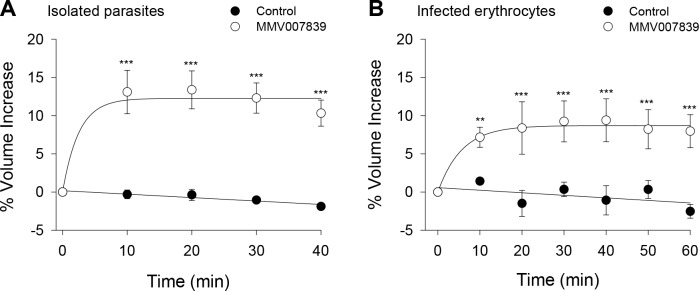
The effect of MMV007839 on the volume of parasites and parasitised erythrocytes. MMV007839 (1 μM) and DMSO (0.1% v/v; solvent control) were tested for their effects on the volume of (A) saponin-isolated 3D7 *P*. *falciparum* trophozoites suspended at 37°C in pH 7.10 Experimental Saline Solution and (B) intact erythrocytes infected with 3D7 trophozoites suspended at 37°C in a pH 7.40 bicarbonate-free RPMI medium (containing supplements as outlined in the Materials and Methods). The data are expressed as a percentage volume increase relative to the starting volume, which for isolated parasites (A) ranged from 35 fL to 46 fL in the individual experiments, and for intact infected erythrocytes (B) ranged from 73 fL to 79 fL. In both cases the data shown are the mean ± SEM from three independent experiments performed on different days. For each condition, the (pre-normalised) volume data for each time point greater than 0 was tested for statistical significance compared to the time zero data for that condition; **P < 0.01, ***P < 0.001.

The MMV007839-induced accumulation of lactate (and glycolytic intermediates) within the parasite might be expected to induce cell swelling, through osmotic effects. To test this we investigated the effect of MMV007839 on the volume of both isolated (3D7) parasites and intact parasitised erythrocytes, using a Coulter Multisizer. Addition of MMV007839 (1 μM) to a suspension of isolated parasites caused the parasites to swell, increasing their volume by 13.1 ± 2.8% (relative to their starting volume; equating to a volume increase of 5.1 ± 0.8 fL; mean ± SEM; n = 3; *P* < 0.001) within 10 min and maintaining a similar volume for the next 30 min (Fig 6A). MMV007839 (1 μM) also caused erythrocytes infected with 3D7 trophozoites to swell, increasing their volume by 7.2 ± 1.3% (relative to their starting volume; equating to a volume increase of 5.4 ± 1.0 fL; mean ± SEM; n = 3; *P* < 0.01) within 10 min and maintaining a similar volume for the next 50 min (Fig 6B).

## Discussion

In this study we provide multiple lines of evidence that two structurally-related compounds from the MMV Malaria Box, MMV007839 and MMV000972, kill asexual blood-stage *P*. *falciparum* parasites via inhibition of the lactate:H^+^ transporter PfFNT. Compounds with this mechanism of action have not been described or exploited as antimalarial drugs previously. Our findings highlight the potential of PfFNT as an antimalarial drug target.

The most direct line of evidence that the two compounds target PfFNT comes from experiments in which PfFNT was studied in isolation from other *P*. *falciparum* transporters in the *Xenopus* oocyte. In this heterologous expression system, MMV007839 and MMV000972 inhibited L-[^14^C]lactate transport via the native form of PfFNT with IC_50_ values of 23 nM and 50 nM, respectively, while not inhibiting two other *P*. *falciparum* transporters (PfCRT and PfENT1). The IC_50_ value for PfFNT inhibition by MMV007839 is somewhat lower than the IC_50_ values we obtained for parasite growth inhibition by this compound (260 nM for 3D7 parasites and 140 nM for Dd2 parasites). The IC_50_ value for parasite growth inhibition by MMV000972 (1.8 μM for Dd2 parasites) was much higher than that obtained for inhibition of native-PfFNT-mediated L-[^14^C]lactate transport. MMV007839 and MMV000972 have also been tested against 3D7 parasites by other groups, yielding IC_50_ values of 283–442 nM and 2.6 μM, respectively (http://www.mmv.org/research-development/malaria-box-supporting-information). Given that MMV007839 and MMV000972 inhibit PfFNT with comparable potency when expressed in oocytes, the much higher IC_50_ value for parasite growth inhibition by MMV000972 compared to MMV007839 might result from a lower concentration of the former compound in the parasitised erythrocyte or the parasite itself.

Parasites selected for resistance to growth-inhibition by MMV007839 had a Gly107Ser mutation in PfFNT. This mutation, which was observed in two independent cultures within six weeks of first adding the compound, decreased the susceptibility of parasites to MMV007839 and MMV000972 by > 30-fold while not affecting their sensitivity to chloroquine or artemisinin. The fact that the MMV007839-selected parasites were cross-resistant to MMV000972 was not surprising in light of the high degree of structural similarity between the compounds and our finding that they both inhibit PfFNT.

The fact that we were able to generate parasites with high-level resistance to MMV007839 and MMV000972 within six weeks is a potential cause for concern with regard to the potential of such compounds as antimalarials. However, this would not necessarily deter further investigations into exploiting PfFNT as a drug target. Many compounds for which resistance has been generated *in vitro* are undergoing development [3], such as the protein translation inhibitor DDD498 [30] and various compounds that have been proposed to target PfATP4 [31–33]. A relationship between the propensity of parasites to develop resistance *in vitro* and the likelihood of parasite resistance emerging *in vivo* is not firmly established. Factors such as the drug’s rapidity of action [34] and the growth rate and transmissibility of drug-resistant parasites also contribute to the overall threat of clinical resistance [35]. Furthermore, carefully selected dosing regimens and partner drugs may reduce the likelihood of selecting for resistance-conferring mutations in the target protein *in vivo*.

Our direct studies of PfFNT inhibition were complemented by *in situ* studies with *P*. *falciparum* parasites. The parasite studies provided multiple lines of evidence (based both on pH measurements and L-[^14^C]lactate flux measurements) that both MMV compounds abolish L-lactate transport across the parasite plasma membrane. Experiments with L-[^14^C]lactate showed that MMV007839 and MMV000972 inhibited the transport of L-lactate across the parasite plasma membrane with IC_50_ values of 158 nM and 49 nM respectively in wild-type Dd2 parasites. These IC_50_ values are similar to those obtained for the inhibition of L-[^14^C]lactate uptake by PfFNT expressed in *Xenopus* oocytes. This suggests that PfFNT may be the only transporter that can mediate the transport of L-lactate across the parasite plasma membrane at an appreciable rate, at least in trophozoite-stage parasites. The parasite does encode two transporters with homology to human monocarboxylate transporters, however neither showed a capacity to transport lactate when expressed heterologously in yeast [17].

The potency with which MMV007839 and MMV000972 inhibited L-[^14^C]lactate transport by parasites was significantly reduced in the resistant parasites bearing the Gly107Ser mutation in PfFNT. Using the *Xenopus* oocyte system, this finding was attributed directly to a reduced inhibitor sensitivity of the mutant PfFNT^Gly107Ser^ protein compared to the native protein. The mutated residue is adjacent to T106, which has been predicted to form part of the PfFNT transport channel [17]. The mutation may reduce the affinity with which MMV007839 and MMV000972 bind to the transporter, or otherwise prevent the compounds from inhibiting its function. The mutation was associated with an approximately 50–60% reduction in L-[^14^C]lactate transport by both parasites and PfFNT expressed in *Xenopus* oocytes. This reduction in lactate transport was not associated with a decrease in the growth rate of asexual blood-stage parasites, suggesting that the rate of lactate export from parasites must be inhibited by more than 50% to affect their viability under the conditions tested here.

The metabolite profiles of MMV007839-treated and untreated parasitised erythrocytes provided further evidence in support of the mechanism of action proposed here. Lactate levels were specifically elevated in MMV007839-treated infected erythrocytes compared to untreated infected erythrocytes, consistent with decreased efflux from infected erythrocytes. The level of lactate secreted into the medium was also reduced in MMV007839-treated cultures relative to untreated cultures (although this decrease did not reach statistical significance), while pyruvate, phosphoenolpyruvate and 3-phosphoglycerate secretion was increased in the MMV007839-treated infected erythrocytes. Collectively, these analyses indicate that MMV007839 induces a build-up of lactate, its immediate precursor (pyruvate), and other glycolytic intermediates. This build-up is partly countered by increased secretion of partly oxidised intermediates and accompanied by significant swelling of both the parasite and the intact infected erythrocyte. The observed accumulation of lactate and pyruvate is a distinctive phenotype that has not been observed previously when infected erythrocytes were treated with a range of antimalarials including glycolysis inhibitors [29]. The metabolic perturbations were observed after 1 h, 3 h and 6 h exposures to MMV007839. As parasite viability was not affected at the early time points, these changes cannot be attributed to cell death.

NADH levels were significantly lower in MMV007839-treated compared to untreated infected erythrocytes. This might be explained by the consumption of NADH in the reaction that produces sn-glycerol 3-phosphate, which was significantly elevated within the MMV007839-treated cells. Significant changes in intracellular abundance were also observed for a range of other metabolites, including a number of (likely haemoglobin-derived) small peptides and pyrimidine precursors. The basis for these changes was not investigated, although they may be a secondary consequence of the MMV007839-induced acidification of the parasite cytosol or disruption to energy metabolism. Previous work has demonstrated that inhibiting the *P*. *falciparum* glucose transporter not only perturbs glycolysis but also impacts other metabolic pathways including pyrimidine biosynthesis and haemoglobin digestion [29]. Studies in which the fates of isotope-labelled metabolites are tracked would be required to resolve the mechanistic basis for the full range of metabolic perturbations resulting from inhibition of PfFNT. Thus while the changes in the levels of glycolytic intermediates observed in MMV007839-treated parasitised erythrocytes are fully consistent with inhibition of PfFNT, it remains possible that changes in the levels of other metabolites could have resulted from off-target effects of MMV007839 rather than as a consequence of downstream effects of PfFNT inhibition.

A recently published study in which extensive toxicity testing was performed on the Malaria Box compounds revealed that MMV007839 and MMV000972 display toxicity in some assays [36]. The compounds themselves are therefore unlikely to be suitable as clinical candidates. Further screening for compounds that inhibit PfFNT will be required to develop suitable clinical candidates that might be used to exploit this target in the field.

In summary, our screen of the Malaria Box for compounds that perturb the pH inside the parasite led to the discovery of two compounds that kill parasites via inhibition of a novel target, PfFNT. The mechanisms of action of the remaining pH-decreasing hits are yet to be determined, and further studies on these compounds may uncover additional targets. Another transport protein that mediates the translocation of H^+^ across the plasma membrane, PfATP4, has emerged recently as a particularly vulnerable drug target [37], and compounds believed to target this pump give rise to a cytosolic alkalinisation. Thus, pH assays can be used as a primary screen for the identification of additional compounds that inhibit either PfFNT or PfATP4.

## Materials and Methods

### Compounds

The Malaria Box [8] was provided by MMV. Upon receipt of the Malaria Box, each compound was diluted to 1 mM in DMSO and aliquoted into multiple plates. Information about the compounds is accessible via the ChEMBL-NTD database (https://www.ebi.ac.uk/chemblntd). Additional quantities of MMV007839 and MMV000972 were obtained from MMV and Vitas-M Laboratory, respectively.

### Ethics statement

The use of human blood in this study was approved by the Australian National University Human Research Ethics Committee. The blood was provided by the Australian Red Cross Blood Service without disclosing the identities of the donors.

Ethical approval of the work performed with the *Xenopus laevis* frogs was obtained from the Australian National University Animal Experimentation Ethics Committee (Animal Ethics Protocol Number A2013/13) in accordance with the Australian Code of Practice for the Care and Use of Animals for Scientific Purposes.

### Parasite culture and isolation

The 3D7 strain of *P*. *falciparum* (isolated in the Netherlands but likely to be of African origin) was used throughout this study, except for the MMV007839 resistance selection studies, which were initiated with a clone of Dd2 (Thai origin) that had been generated previously by limiting dilution [38], and the subsequent experiments performed to characterise the resistant parasites. The 3D7 strain and the Dd2 clone were checked for mycoplasma contamination prior to their use in this study and were found to be mycoplasma-free. Parasites were cultured in human erythrocytes [39], with continuous shaking [40], and were synchronised by sorbitol treatment [41]. The culture medium was RPMI 1640 containing 25 mM HEPES (Gibco) supplemented with 11 mM additional glucose, 0.2 mM hypoxanthine, 20 μg/mL gentamicin sulphate and 3 g/L Albumax II.

Prior to cytosolic pH, digestive vacuole pH, parasite volume and L-[^14^C]lactate uptake measurements, mature trophozoite-stage parasites (approximately 34–40 h post-invasion) were functionally isolated from their host erythrocytes by brief exposure (of cultures at approximately 4% haematocrit) to saponin (0.05% w/v, of which ≥ 10% was the active agent sapogenin) [42]. Unless stated otherwise the parasites were then washed several times in bicarbonate-free RPMI 1640 supplemented with 11 mM additional glucose, 0.2 mM hypoxanthine and 25 mM HEPES (pH 7.10), and maintained in this medium at a density of 1 × 10^7^–3 × 10^7^ parasites mL^-1^ at 37°C until immediately before their use in experiments.

### Measurement of the cytosolic pH in isolated parasites

Cytosolic pH was measured as described previously [25] at 37°C (unless stated otherwise) using saponin-isolated trophozoites loaded with the pH-sensitive fluorescent dye BCECF (Molecular Probes). In most experiments parasites were suspended in ‘Experimental Saline Solution’ (125 mM NaCl, 5 mM KCl, 1 mM MgCl_2_, 20 mM glucose, 25 mM HEPES; pH 7.10 unless stated otherwise). For experiments in which parasites were deprived of glucose, glucose-free Experimental Saline Solution was used (135 mM NaCl, 5 mM KCl, 1 mM MgCl_2_, 25 mM HEPES; pH 7.10). Parasites suspended at a density of 1 × 10^7^–3 × 10^7^ parasites mL^-1^ were excited successively at 440 and 495 nm, with emission recorded at 520 nm, using either a PerkinElmer LS 50B Fluorescence Spectrometer or a Cary Eclipse Fluorescence Spectrophotometer. The relationship between the ratio of the two measurements (495 nm/440 nm) and the cytosolic pH was calibrated as described previously [25] with one exception: for experiments performed at 4°C, the previously published calibration method yielded abnormally high values for resting pH, and the calibration was therefore performed using Experimental Saline Solution (adjusted to various pH values) in place of the high-K^+^ calibration solutions.

### Measurement of pH in the parasite’s digestive vacuole

The pH inside the digestive vacuole (pH_DV_) was monitored at 37°C using isolated parasites containing fluorescein-dextran (10,000 MW; Molecular Probes) in their digestive vacuoles. The parasites were prepared as described previously [43] and suspended in Experimental Saline Solution (pH 7.10) at a density of 1 × 10^7^–3 × 10^7^ parasites mL^-1^. Fluorescence ratio measurements were obtained with a PerkinElmer Life Sciences LS 50B fluorometer with a dual excitation Fast Filter accessory using excitation wavelengths of 440 and 495 nm and an emission wavelength of 520 nm [44].

### Measurements of L-[^14^C]lactate uptake by parasites

The uptake of L-[^14^C]lactate (PerkinElmer) by isolated 3D7 trophozoites was determined essentially as described previously [16]. Experiments were performed at 4°C on parasites suspended in pH 6.10 Experimental Saline Solution. Parasites were preincubated with either DMSO or inhibitors (at the concentrations stated in the relevant Figure legends) for 1 min before a 200 μL sample of the suspension was added (at ‘time zero’) to an equal volume of pH 6.10 saline (with or without inhibitors as specified in the Figure legends) layered over a 200 μL dibutyl phthalate/dioctyl phthalate (5:4 v/v) oil mix (in a microcentrifuge tube) and containing L-[^14^C]lactate (giving a final concentration after the cell suspension was added of 1.3 μM).

At 20 s after combining the parasites and L-[^14^C]lactate, uptake was terminated by centrifuging the sample (15,800 × *g*, 2 min in a rapid-acceleration Beckman Microfuge E), thereby sedimenting the parasites below the oil layer. A 10 μL aliquot of the supernatant solution was taken from above the oil layer in each tube to enable the determination of extracellular L-[^14^C]lactate concentrations. The remaining supernatant solution was discarded and residual radioactivity on the sides of each tube was removed by rinsing the tube four times with water before aspirating most of the oil. The cell pellets were lysed using Triton X-100 (0.1% v/v) and deproteinised with trichloroacetic acid (2.5% w/v final) before the radioactivity in each sample was measured using a scintillation counter [42].

In each experiment the amount of radioactivity in the cell pellets that was attributable to ‘trapped’ extracellular L-[^14^C]lactate was estimated under conditions in which the amount of L-[^14^C]lactate taken up by the parasites was minimised by using the previously-described [16] lactate transport inhibitor NPPB. Parasites were preincubated with NPPB (100 μM) for 1 min then combined with a L-[^14^C]lactate solution to which unlabelled L-lactate and NPPB had been added (yielding a final lactate concentration of 10 mM and a final NPPB concentration of 100 μM), then centrifuged immediately.

‘Distribution ratios’ (the intracellular L-[^14^C]lactate concentration divided by the extracellular L-[^14^C]lactate concentration) were calculated as described previously [16]. The extracellular L-[^14^C]lactate concentration for each sample was determined using the aliquots of supernatant solution taken from above the oil layer. The intracellular L-[^14^C]lactate concentration for each sample was determined from the radioactivity incorporated into the cell pellet (after subtraction of the radioactivity attributable to the trapped extracellular L-[^14^C]lactate), the parasite number, and the previously determined estimate of the water volume of a saponin-isolated parasite [42].

### Parasite proliferation assays

Parasite proliferation was measured in 96-well plates using a fluorescent DNA-intercalating dye [45]. The assays were initiated with erythrocytes infected with predominantly ring-stage parasites, and the starting parasitaemia was 0.5–1% and the haematocrit was 1%. The experimental protocol and procedure for data analysis were the same as those outlined previously [46] with two modifications: the duration of the assay was 68–72 h, and a supramaximal concentration of an antiplasmodial compound (either 5 μM chloroquine, 0.5 μM artemisinin, 12.8 nM KAE609 or 150 μM MMV007839) was used for the ‘zero growth’ control. The concentration of DMSO in the assays did not exceed 0.2% (v/v).

### Measurements of the volume of parasites and infected erythrocytes

The volume of saponin-isolated 3D7 trophozoites and of erythrocytes infected with 3D7 trophozoites was measured using a Beckman Coulter Multisizer 4 fitted with a 100 μm ‘aperture tube’. In the case of isolated parasites, the cells were washed and resuspended (at 37°C) in pH 7.10 Experimental Saline Solution. The electrolyte solution within the aperture tube was the same as the Experimental Saline Solution except that it lacked glucose. For experiments on infected erythrocytes, the infected cells were separated from uninfected erythrocytes using a Miltenyi Biotec VarioMACS Magnet [47, 48] and the cells were maintained at 37°C in bicarbonate-free RPMI supplemented with 25 mM HEPES, additional glucose (11 mM) and 0.2 mM hypoxanthine, and pH-adjusted to 7.40. For these measurements the electrolyte solution within the aperture tube differed only from this medium in that it was not supplemented with hypoxanthine or additional glucose. For each measurement of cell volume, approximately 20,000 pulses (each corresponding to the passage of a single cell through the aperture) were recorded. The median volume of the cells within each sample was determined by fitting a log Gaussian distribution curve to the population data.

### Generating MMV007839-resistant parasites and sequencing *PfFNT*

A clone of the chloroquine-resistant Dd2 strain of *P*. *falciparum* was used to generate MMV007839-resistant parasites. This clone was generated previously by limiting dilution and was used successfully to generate resistance to two (PfATP4-associated) Malaria Box compounds [18]. Two independent cultures (each containing ~ 5 × 10^8^ parasites) were exposed to increasing concentrations of MMV007839 for ~ 6 weeks, starting with a concentration of 100 nM on Day 0, and increasing to 125 nM (approximately the IC_50_ value for the parental Dd2 line) on Day 3. For MMV007839-selected culture A, the drug concentration was increased by 5 nM increments on Day 18, Day 21, and every two days thereafter. For MMV007839-selected culture B, the drug concentration was increased by 15 nM increments every two days starting on Day 18. The cultures were provided with fresh medium and blood and diluted to reduce the parasitaemia as needed.

Genomic DNA was extracted from saponin-isolated parasites (from the MMV007839-selected bulk cultures) using a QIAGEN Plant DNeasy kit. To sequence exons 1–3, the entire *PfFNT* gene was PCR-amplified using KOD Hot Start DNA polymerase and primers 1 and 2 (S2 Table). The PCR product was extracted using a GeneJET Gel Extraction Kit (Thermo Fisher Scientific). Sequencing was performed at the ACRF Biomolecular Resource Facility (The John Curtin School of Medical Research, Australian National University) using primers 1 and 3–7 (S2 Table). To sequence the fourth (and final) exon, the 3’ end of the gene and part of the 3’ UTR was PCR amplified using the primers 3 and 8 (S2 Table), with primer 8 also used for sequencing.

For fitness assays, two clones were obtained from MMV007839-selected culture B by limiting dilution, performed essentially as described previously [38]. The presence of the G319A mutation in the *PfFNT* coding sequence (giving rise to the Gly107Ser mutation in PfFNT) was confirmed in both clones.

### Metabolite extraction and LC-MS analyses

Erythrocytes infected with 3D7 trophozoites were separated from uninfected erythrocytes using a magnet supplied by Colebrook Bioscience [47, 48]. The resulting cells, which consisted of > 95% infected erythrocytes, were then allowed to recover for 0.5–1 h at 37°C in ‘complete medium’ (RPMI 1640 supplemented with 0.5% Albumax II, 25 mM HEPES, 100 μM hypoxanthine and 10 μg/mL gentamycin). At ‘time zero’, MMV007839 (6 μM) was added to three cell suspensions (each containing 1 × 10^8^ cells at 0.4% haematocrit), while three identical cell suspensions were left untreated. The cell suspensions were incubated at 37°C under controlled atmospheric conditions (5% CO_2_ and 1% O_2_ in N_2_). At each of three time points (1 h, 3 h and 6 h), one MMV007839-treated cell suspension and one untreated cell suspension were used to extract metabolites. To extract metabolites, cell suspensions were first pelleted by centrifugation (14,000 × *g*, 30 s). Aliquots (5 μL) of the supernatant media were collected and the remaining supernatant media discarded. The cells were then washed with 1 mL of ice-cold PBS. To extract metabolites, 200 μL volumes of 80% acetonitrile (in H_2_O; containing 5 μM [^13^C]aspartate as an internal standard) were added to the cell pellets and samples of extracellular media. The samples were vortexed briefly then centrifuged (14,000 × *g*, 10 min, 4°C), and the resulting supernatant solutions were transferred to vials for LC-MS analysis.

The metabolites were separated on a SeQuant ZIC-pHILIC column (5 μm, 150 × 4.6 mm, Millipore) with a 1200 series HPLC system (Agilent), using a flow rate of 0.3 mL/min with 20 mM ammonium carbonate in water (A) and 100% acetonitrile (B) as the mobile phase. A binary gradient was set up as follows: 0.5 min: 20% A and 80% B, 30 min: 80% A and 20% B, 31 min: 95% A and 5% B, 35.5–45 min: 20% A and 80% B. Detection of metabolites was performed on an Agilent Q-TOF mass spectrometer 6550 operating in negative ESI mode. The scan range was 85–1200 m/z between 5 and 35 min at 0.9 spectra/second.

LC-MS.d files were converted to.mzXML files using MS convert and analysed using MAVEN [49]. Following alignment, metabolites were assigned using exact mass (< 5 ppm) and retention time (compared to a standards library of 150 compounds run on the same day). The area top for each positively assigned metabolite was integrated and used to calculate the ratio of the metabolite concentration between MMV007839-treated and untreated controls. Log_2_ ratios (+ MMV007839/- MMV007839) across the time series were then plotted using the heatmap script in R.

### Experiments with *Xenopus laevis* oocytes

The *Xenopus laevis* oocyte expression system was used to assess the effects of MMV007839 and MMV000972 on PfFNT (from the 3D7 strain), PfENT1 (from the FAF6 strain) and PfCRT (from the Dd2 strain). The oocyte expression vectors containing *PfFNT*, *PfENT1* and *PfCRT* were made previously [16, 26, 50]. The Gly107Ser mutant form of PfFNT was generated through site-directed mutagenesis of the oocyte expression vector containing wild-type (3D7) *PfFNT* using primers 9 and 10 (S2 Table). Oocytes were harvested from adult female *Xenopus laevis* frogs and prepared as described previously [51]. cRNA was made using the mMessage mMachine T7 transcription kit and the MEGAclear kit (Ambion) and microinjected into oocytes as outlined elsewhere [51]. The amount of cRNA injected (per oocyte) was 30 ng for PfFNT, 10 ng for PfENT1, and 20 ng for PfCRT. The uptake of radiolabelled substrates was measured 2–5 days post-injection at 27.5°C in ND96 buffer (containing 96 mM NaCl, 2 mM KCl, 1 mM MgCl_2_, 1.8 mM CaCl_2_, 10 mM MES and 10 mM Tris-base; pH-adjusted to the value specified in the relevant Figure legends). L-[^14^C]lactic acid (Na^+^ salt; 150.6 mCi/mmol) and [^3^H]hypoxanthine monochloride (14 Ci/mmol) were purchased from Perkin Elmer, and [^3^H]chloroquine (20 Ci/mmol) was purchased from American Radiolabeled Chemicals. Each experiment used ten oocytes for each condition tested. The influx of the radiolabelled substrate was halted by removing the reaction buffer and washing the oocytes twice in ice-cold ND96 buffer (3.5 mL). The oocytes were then transferred to separate wells of a 96-well plate, lysed with 10% SDS, and the radioactivity measured as described previously [52].

### Statistics

For measurements of cell volume, one-way ANOVAs were carried out with the pre-normalised data using ‘experiment’ as a ‘blocking factor’, to prevent differences in the starting volume between independent experiments (which may have resulted from differences in the average age of the parasites on the different days) from eroding the precision of the test. *Post hoc* comparisons were then performed using the least significant difference test.

For the LC-MS data, two-way ANOVAs were performed for both the extracellular and intracellular metabolite datasets. Between-subject testing was performed and the results corrected for multiple hypothesis testing using the False Discovery Rate determined by MetaboAnalyst [53].

For all other comparisons, *P* values were obtained using either one-way ANOVAs followed by *post hoc* Tukey tests or t-tests (paired or unpaired as appropriate), as stated in the relevant sections. All tests were two-sided.

## Supporting Information

S1 Table The Malaria Box ‘hits’ from *P*. *falciparum* pH assays and their chemical structures. (PDF)

S2 Table Primers used in this study. (PDF)

S1 Fig Fluorescence traces showing the effects of two Malaria Box compounds on the pH inside the digestive vacuole. Representative traces showing the effect of (A) the addition of a Malaria Box compound that had an effect on the pH inside the digestive vacuole (pH_DV_; 1 μM MMV665807), and (B) the addition of a compound from S1 Table that was without effect on pH_DV_ (1 μM MMV666080), followed by the addition of the V-type H^+^-ATPase inhibitor concanamycin A (conA; 100 nM). The experiments were performed at 37°C with isolated 3D7 trophozoite-stage parasites in which the digestive vacuole had been preloaded with the pH-sensitive probe fluorescein dextran. The parasites were suspended in Experimental Saline Solution (pH 7.10). An increase in fluorescence ratio is indicative of an increase in pH_DV_.
(TIF)

S2 Fig L-[^14^C]lactate influx into 3D7 parasites. The uptake of L-[^14^C]lactate into isolated 3D7 *P*. *falciparum* trophozoites was determined after a 1 min pre-incubation with DMSO (0.4% v/v; solvent control), NPPB (a non-specific anion transport inhibitor; 100 μM), and MMV007839 and MMV000972 (1 μM and 4 μM). The experiment was performed at 4°C in pH 6.1 Experimental Saline Solution, with the uptake of L-[^14^C]lactate measured 20 s after its addition. The concentrations of DMSO, NPPB, MMV007839 and MMV000972 during the 20 s uptake were half of those present during the initial 1 min incubation (the final concentrations were as indicated on the Figure). The mean distribution ratio (intracellular L-[^14^C]lactate concentration/extracellular L-[^14^C]lactate concentration) and SEM from four independent experiments are shown. Data for each condition were tested for statistical significance compared to the control using one-way ANOVAs with *post hoc* Tukey tests; ***P < 0.001.
(TIF)

S3 Fig Analysis of the relative growth rates of MMV007839-resistant parasites (with a Gly107Ser mutation in PfFNT) and their parental parasites. The two panels show the results obtained for two PfFNT^Gly107Ser^-mutant parasite clones, both obtained from MMV007839-selected culture B. Parent and mutant parasites were mixed in approximately equal proportions on Day 0. The different sensitivities of the mutant and parental parasites to MMV007839 enabled their relative proportions to be monitored in the mixed cultures. The (MMV007839-resistant) mutant clones had no growth advantage or disadvantage relative to their parent, with the relative proportions of the mutant and parental parasites remaining similar over three weeks in both cases. The data for the mixed cultures on Day 0 and on Week 3 (measured between Day 20 and Day 25) are the mean + or—SEM from four independent competition experiments. The data for the pure cultures are the mean ± SEM from seven to eight independent experiments performed in parallel with the mixed cultures at various time points throughout the competition experiments, and in the case of the Dd2 parent are the same for Panels A and B.
(TIF)

S4 Fig The effects of MMV007839 and MMV000972 on L-[^14^C]lactate uptake by *Xenopus* oocytes expressing native PfFNT or PfFNT^Gly107Ser^. The uptake of L-[^14^C]lactate by oocytes expressing native PfFNT (black symbols; A and C), oocytes expressing PfFNT^Gly107Ser^ (white symbols; B and D), and non-injected oocytes (grey symbols; all panels) was measured in the presence of a range of different concentrations of MMV007839 (A and B) and MMV000972 (C and D). The data are from the experiments for which data are shown in Fig 4, but are presented here as total L-[^14^C]lactate uptake (expressed as a percentage of that measured for native-PfFNT-expressing oocytes in the absence of MMV compound) rather than PfFNT-mediated L-[^14^C]lactate uptake. I.e., the uptake of L-[^14^C]lactate by non-injected oocytes is shown here and has not been subtracted from that measured in the PfFNT-expressing oocytes.
(TIF)

S5 Fig The effect of MMV007839 and MMV000972 on [^3^H]chloroquine and [^3^H]hypoxanthine uptake by *Xenopus* oocytes expressing PfCRT or PfENT1. MMV007839 and MMV000972 were tested (both at 1 μM and 4 μM) for their effects on (A) the uptake of [^3^H]chloroquine by non-injected oocytes (white bars) and by oocytes expressing PfCRT (from the chloroquine-resistant Dd2 strain; black bars) and (B) the uptake of [^3^H]hypoxanthine by non-injected oocytes (white bars) and by oocytes expressing PfENT1 (black bars). In A, the uptake of [^3^H]chloroquine (0.25 μM) was measured over 1.5–2 h in the presence of 15 μM unlabelled chloroquine at pH 5.5. Verapamil (VP; an inhibitor of [^3^H]chloroquine transport by the Dd2 isoform of PfCRT [26] and added here at a concentration of 40 μM) was included as a control. The data are averaged from four to five independent experiments (performed using oocytes from different frogs) and are shown + SEM. In B, the uptake of [^3^H]hypoxanthine (0.36 μM) was measured over 30 min at pH 6.0. Thymidine (Thy; shown previously to be a substrate for, and hence a competitive inhibitor of, PfENT1 [54], and added here at a concentration of 10 mM) was included as a control. The data are averaged from three to six independent experiments (performed using oocytes from different frogs) and are shown + SEM. Data for each condition were tested for statistical significance compared to the relevant (PfCRT-expressing or PfENT1-expressing) control using one-way ANOVAs with *post hoc* Tukey tests; *P < 0.05, ***P < 0.001. No significant differences were observed in uptake (within the [^3^H]chloroquine or the [^3^H]hypoxanthine datasets) between the non-injected oocyte treatments (one-way ANOVAs with *post hoc* Tukey tests).
(TIF)

S6 Fig The effect of 6 μM MMV007839 on the viability of 3D7 parasites after 1 h, 3 h and 6 h exposures. On Day 0, cultures containing erythrocytes infected with 3D7 trophozoites (as well as uninfected erythrocytes) were exposed to either MMV007839 (6 μM) or DMSO (0.02% v/v; solvent control) for 1 h, 3 h or 6 h. The compound (or DMSO) was then removed by washing the cells three times in culture medium and the cells were diluted with fresh culture medium and supplemented with uninfected erythrocytes. The parasitaemias of the cultures were determined on Day 3. Within each experiment, the parasitaemia obtained for the MMV007839-treated culture was normalised to that of the culture treated with 0.02% v/v DMSO for the same length of time. The data are averaged from three independent experiments and are shown + SEM. The (pre-normalised) data for MMV007839- and DMSO-treated cultures were compared at each time point using paired t-tests; **P < 0.01.
(TIF)

S1 Data LC-MS data and statistical analyses. (XLSX)
