# Supplementary material for: The Malaria Parasite's Lactate Transporter PfFNT Is the Target of Antiplasmodial Compounds Identified in Whole Cell Phenotypic Screens
Source: PLoS Pathog. 2017 Feb 8;13(2):e1006180. doi: 10.1371/journal.ppat.1006180 (PMC5298231; doi:10.1371/journal.ppat.1006180)
Supplement: S1 Table — (PDF) [file ppat.1006180.s001.pdf]

Supplementary Table 1. The Malaria Box 'hits' from *P. falciparum* pH assays and their chemical structures.

Compound	IC_{50} for killing of 3D7 parasites (μM) (from [1])	Structure	Plate	Well	Complete alkalinisation of DV?[a]		Comments
					1 μM	5 μM	
MMV007839	0.28	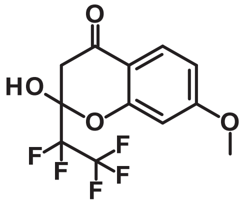	A	H2	No	No	Shown in this study to be a PfFNT inhibitor
MMV007907	0.35	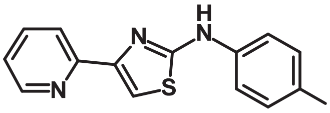	A	H9	No	No	Effect on pH_{cyt} was minor[b] at 1 μM
MMV665909	1.36	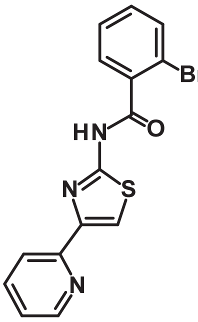	B	B8	No	No	Effect on pH_{cyt} was minor at 1 μM
MMV665807	ND	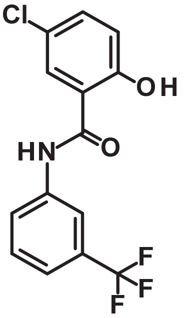	B	C11	Yes	Yes	
MMV666080	1.17	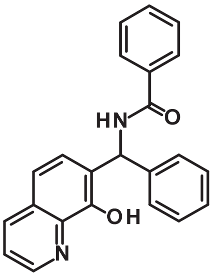	B	F2	No	No	
MMV665953	2.06	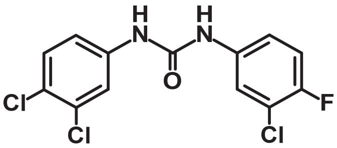	B	G4	No	Yes	

MMV000788	0.70	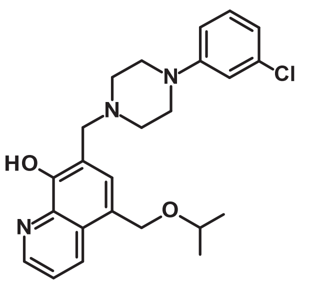	B	H6	No	No	Effect on pH_{cyt} was minor at 1 μM
MMV403679	0.93	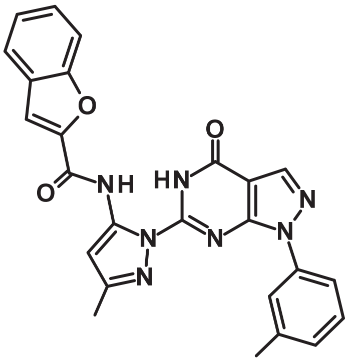	C	A4	No	No	Effect on pH_{cyt} was minor at 1 μM
MMV000972	ND	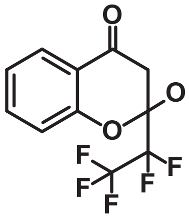	C	E6	No	No	Shown in this study to be a PfFNT inhibitor
MMV665794	0.31	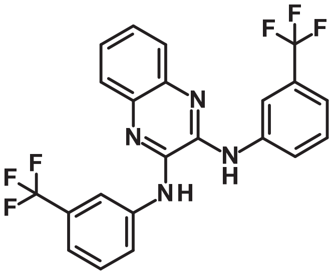	D	C11	Yes	Yes	Effect on pH_{cyt} was minor at 1 μM
MMV665987	0.53	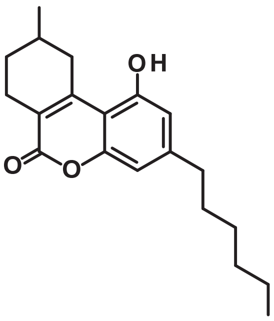	D	D4	No	Yes	
MMV007224	1.06	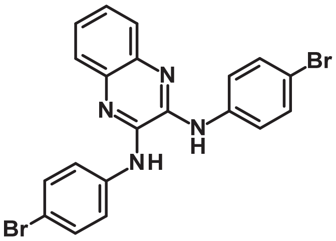	D	H6	No	Yes	Effect on pH_{cyt} was minor at 1 μM

MMV665972	1.14	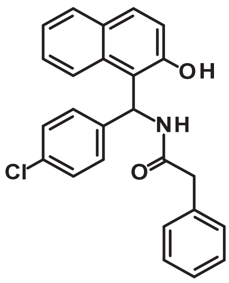	D	H11	No	No	Effect on pH_{cyt} was minor at 1 μM
MMV665852	1.16	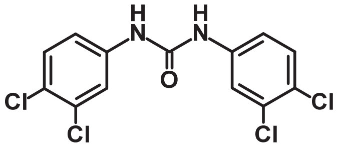	E	E9	No	Yes	
MMV665864	0.71	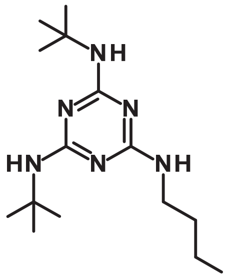	E	F10	No	Yes	

The compounds all gave rise to a decrease in cytosolic pH (pH_{cyt}) when tested at a concentration of 1 μM. Note that the Table does not include the PfATP4-associated hits that were shown to give rise to an *increase* in cytosolic pH in a previous study [2]. The Malaria Box was from the May 2012 batch. ND, not determined.

[a]Effects on digestive vacuole pH (pH_{DV}) were tested using a concentration of 1 μM in one experiment and 5 μM in a separate experiment. The alkalinisation of the digestive vacuole induced by a compound was considered complete if the increase in fluorescence ratio observed was similar in magnitude to that seen on exposure of parasites to a supramaximal concentration (100 nM) of the V-type H^+-ATPase inhibitor concanamycin A [3].

[b]A minor effect on pH_{cyt} corresponds to a change of < 0.15 pH unit.

1. Van Voorhis WC, Adams JH, Adelfio R, Ahyong V, Akabas MH, Alano P, et al. Open source drug discovery with the Malaria Box compound collection for neglected diseases and beyond. PLoS Pathog. 2016;12: e1005763.
2. Lehane AM, Ridgway MC, Baker E, Kirk K. Diverse chemotypes disrupt ion homeostasis in the malaria parasite. Mol Microbiol. 2014;94: 327-339.

3. Saliba KJ, Allen RJ, Zissis S, Bray PG, Ward SA, Kirk K. Acidification of the malaria parasite's digestive vacuole by a H^+-ATPase and a H^+-pyrophosphatase. J Biol Chem. 2003;278: 5605-5612.
