# Supplementary material for: The Malaria Parasite's Lactate Transporter PfFNT Is the Target of Antiplasmodial Compounds Identified in Whole Cell Phenotypic Screens
Source: PLoS Pathog. 2017 Feb 8;13(2):e1006180. doi: 10.1371/journal.ppat.1006180 (PMC5298231; doi:10.1371/journal.ppat.1006180)
Supplement: S2 Table — (PDF) [file ppat.1006180.s002.pdf]

Supplementary Table 2. Primers used in this study.

Primer	Direction	Sequence (5' – 3')
1	Forward	ATGCCACCAAATAATTCCAAATATG
2	Reverse	TCAATTTCGTAATTCTATAGATAAAC
3	Forward	GGTGCTGTATCTTTTGCATTTTTTG
4	Reverse	GCCCACAGCTAATGACACACATTC
5	Forward	GTAGGAGCATCGATGAGTGTG
6	Reverse	CTGCTATTCCGGAAGCGTGTG
7	Forward	GATTGCTGGTGCAATTGTTTTGG
8	Reverse	CACCTGGGATAATTAAATAATC
9	Forward	CAGGTTCTGATTTGTTTACGAGTAATACTTTAGCAGTAACC
10	Reverse	GGTTACTGCTAAAGTATTACTCGTAAACAAATCAGAACCTG
