# Supplementary figures and images for: The Malaria Parasite's Lactate Transporter PfFNT Is the Target of Antiplasmodial Compounds Identified in Whole Cell Phenotypic Screens
Source: PLoS Pathog. 2017 Feb 8;13(2):e1006180. doi: 10.1371/journal.ppat.1006180 (PMC5298231; doi:10.1371/journal.ppat.1006180)

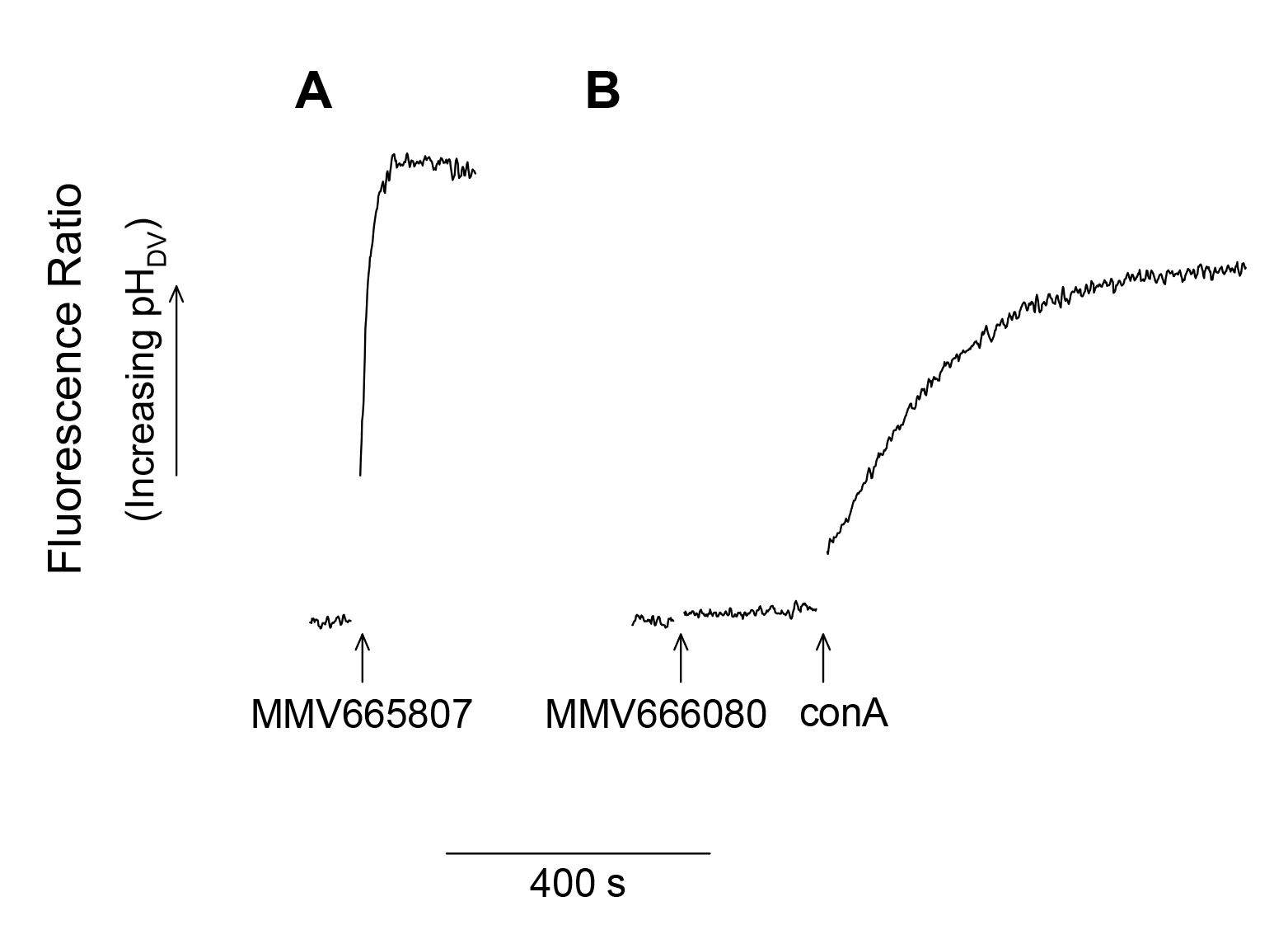

Supplement: S1 Fig — Representative traces showing the effect of (A) the addition of a Malaria Box compound that had an effect on the pH inside the digestive vacuole (pHDV; 1 μM MMV665807), and (B) the addition of a compound from S1 Table that was without effect on pHDV (1 μM MMV666080), followed by the addition of the V-type H+-ATPase inhibitor concanamycin A (conA; 100 nM). The experiments were performed at 37°C with isolated 3D7 trophozoite-stage parasites in which the digestive vacuole had been preloaded with the pH-sensitive probe fluorescein dextran. The parasites were suspended in Experimental Saline Solution (pH 7.10). An increase in fluorescence ratio is indicative of an increase in pHDV. (TIF) [file ppat.1006180.s003.tif]

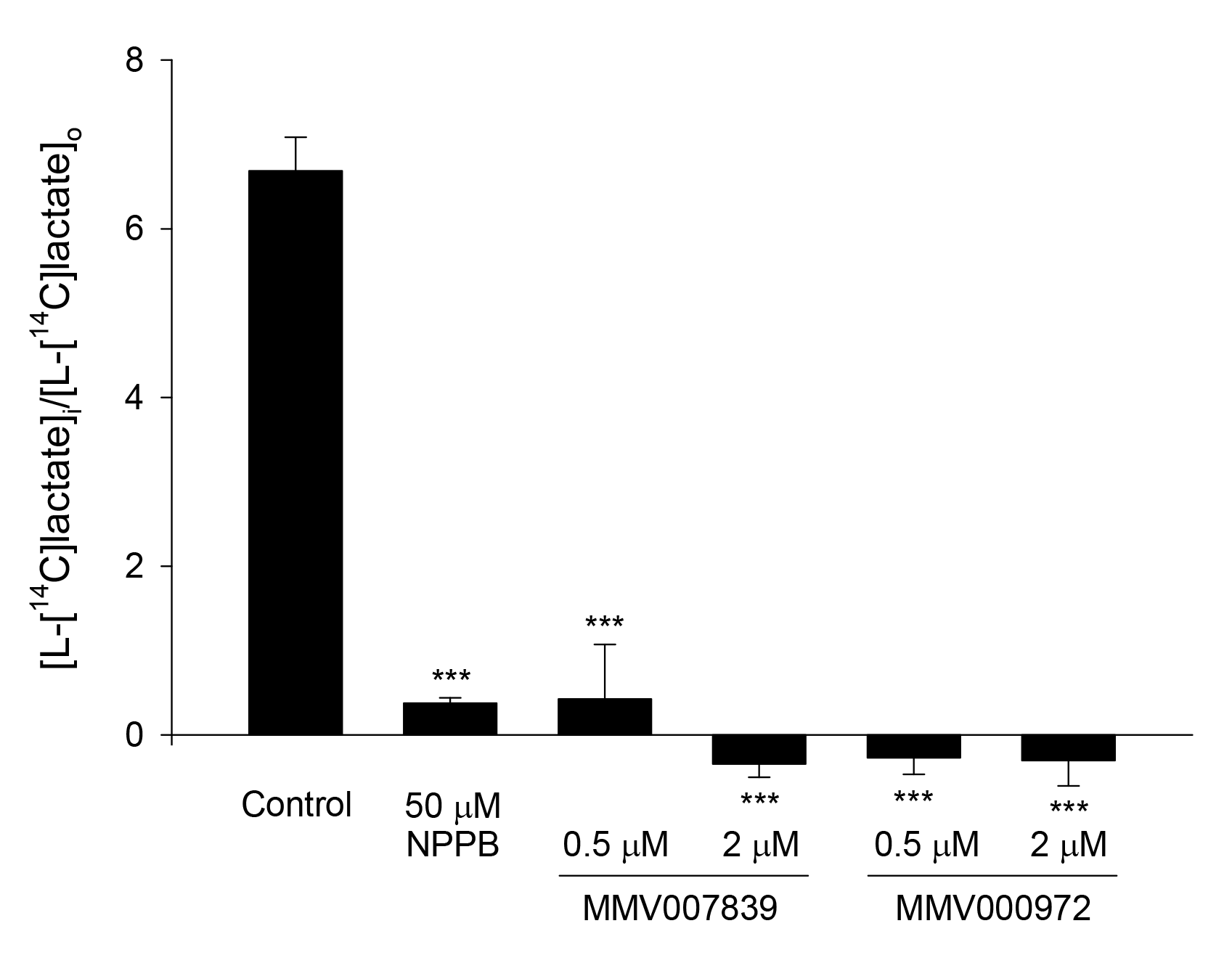

Supplement: S2 Fig — The uptake of L-[14C]lactate into isolated 3D7 P. falciparum trophozoites was determined after a 1 min pre-incubation with DMSO (0.4% v/v; solvent control), NPPB (a non-specific anion transport inhibitor; 100 μM), and MMV007839 and MMV000972 (1 μM and 4 μM). The experiment was performed at 4°C in pH 6.1 Experimental Saline Solution, with the uptake of L-[14C]lactate measured 20 s after its addition. The concentrations of DMSO, NPPB, MMV007839 and MMV000972 during the 20 s uptake were half of those present during the initial 1 min incubation (the final concentrations were as indicated on the Figure). The mean distribution ratio (intracellular L-[14C]lactate concentration/extracellular L-[14C]lactate concentration) and SEM from four independent experiments are shown. Data for each condition were tested for statistical significance compared to the control using one-way ANOVAs with post hoc Tukey tests; ***P < 0.001. (TIF) [file ppat.1006180.s004.tif]

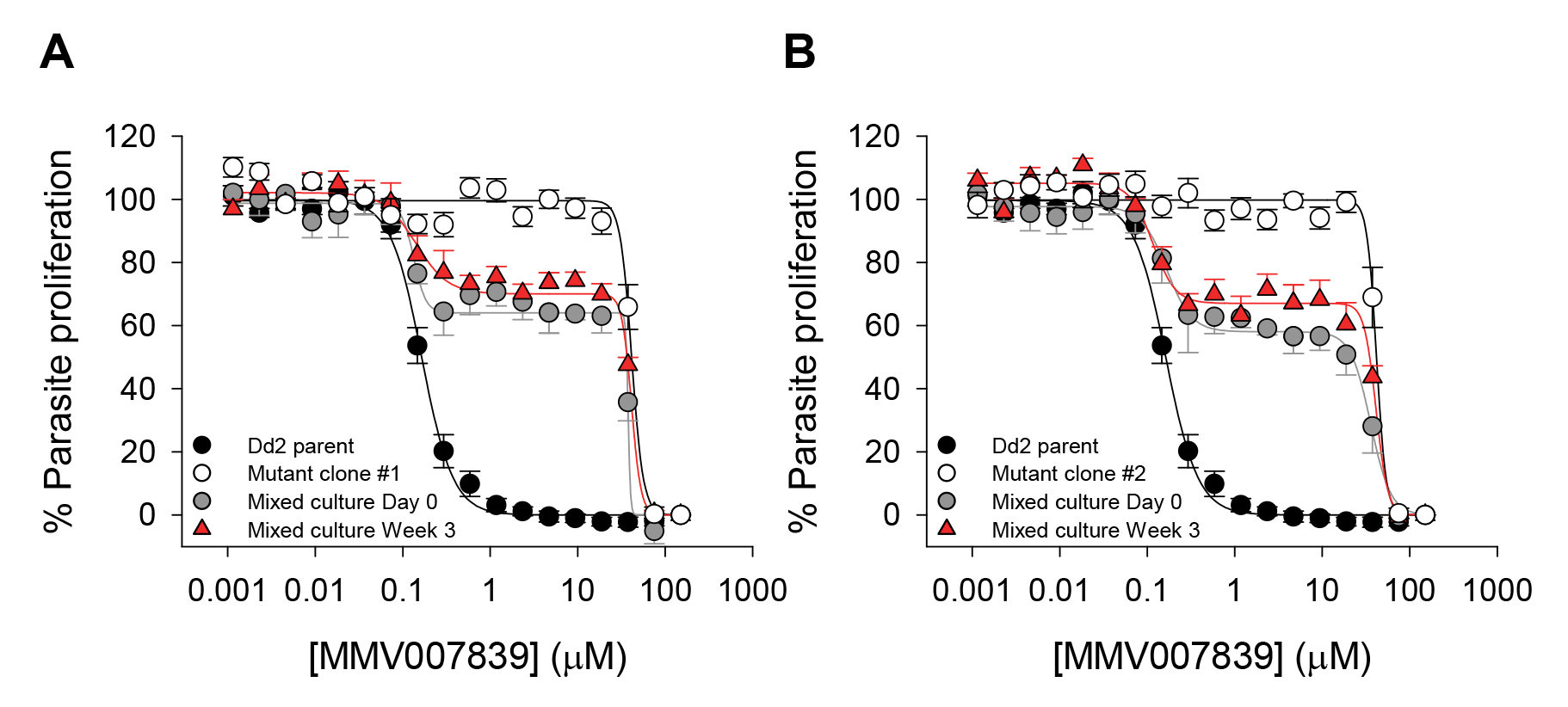

Supplement: S3 Fig — The two panels show the results obtained for two PfFNTGly107Ser-mutant parasite clones, both obtained from MMV007839-selected culture B. Parent and mutant parasites were mixed in approximately equal proportions on Day 0. The different sensitivities of the mutant and parental parasites to MMV007839 enabled their relative proportions to be monitored in the mixed cultures. The (MMV007839-resistant) mutant clones had no growth advantage or disadvantage relative to their parent, with the relative proportions of the mutant and parental parasites remaining similar over three weeks in both cases. The data for the mixed cultures on Day 0 and on Week 3 (measured between Day 20 and Day 25) are the mean + or—SEM from four independent competition experiments. The data for the pure cultures are the mean ± SEM from seven to eight independent experiments performed in parallel with the mixed cultures at various time points throughout the competition experiments, and in the case of the Dd2 parent are the same for Panels A and B. (TIF) [file ppat.1006180.s005.tif]

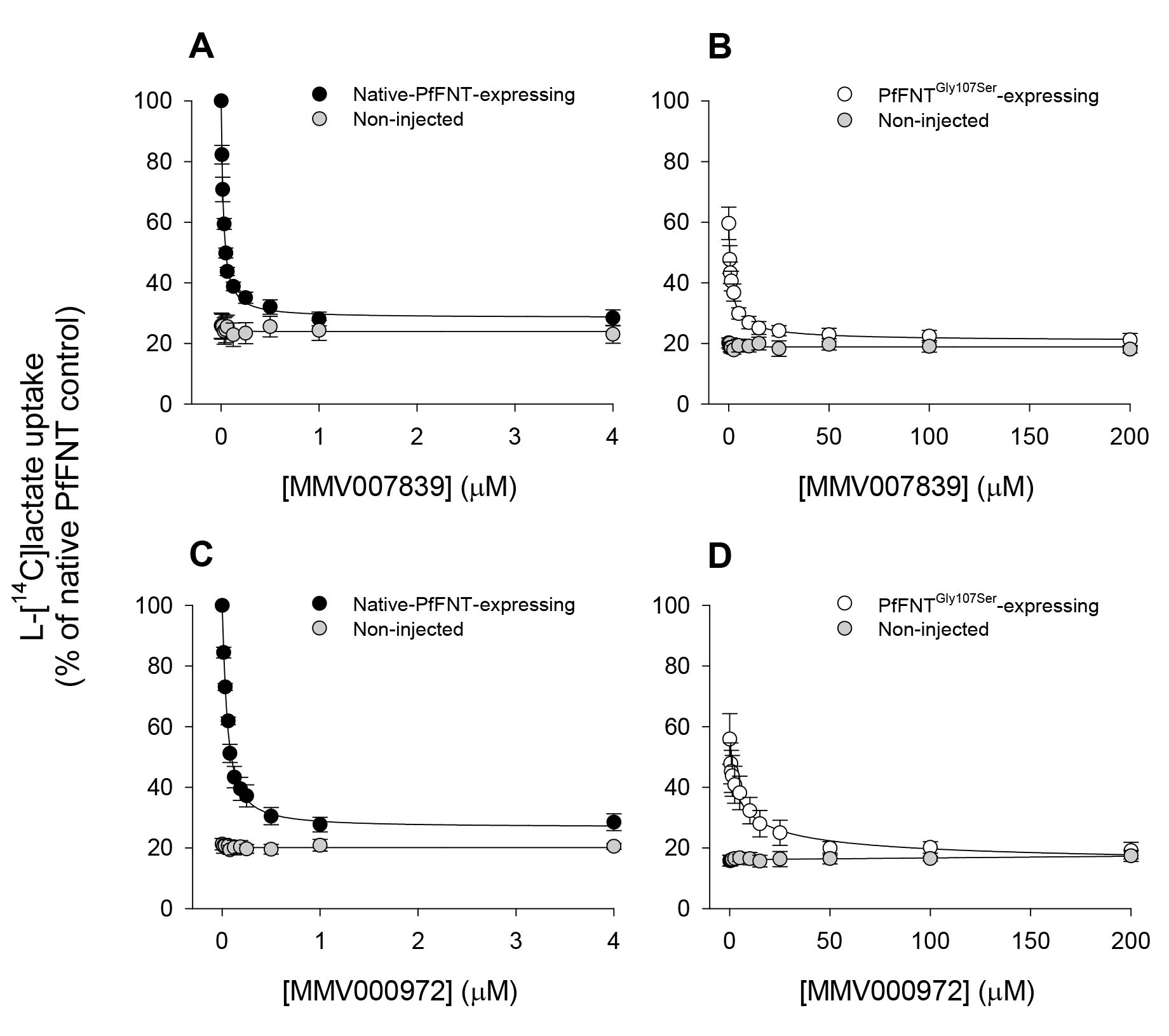

Supplement: S4 Fig — The uptake of L-[14C]lactate by oocytes expressing native PfFNT (black symbols; A and C), oocytes expressing PfFNTGly107Ser (white symbols; B and D), and non-injected oocytes (grey symbols; all panels) was measured in the presence of a range of different concentrations of MMV007839 (A and B) and MMV000972 (C and D). The data are from the experiments for which data are shown in Fig 4, but are presented here as total L-[14C]lactate uptake (expressed as a percentage of that measured for native-PfFNT-expressing oocytes in the absence of MMV compound) rather than PfFNT-mediated L-[14C]lactate uptake. I.e., the uptake of L-[14C]lactate by non-injected oocytes is shown here and has not been subtracted from that measured in the PfFNT-expressing oocytes. (TIF) [file ppat.1006180.s006.tif]

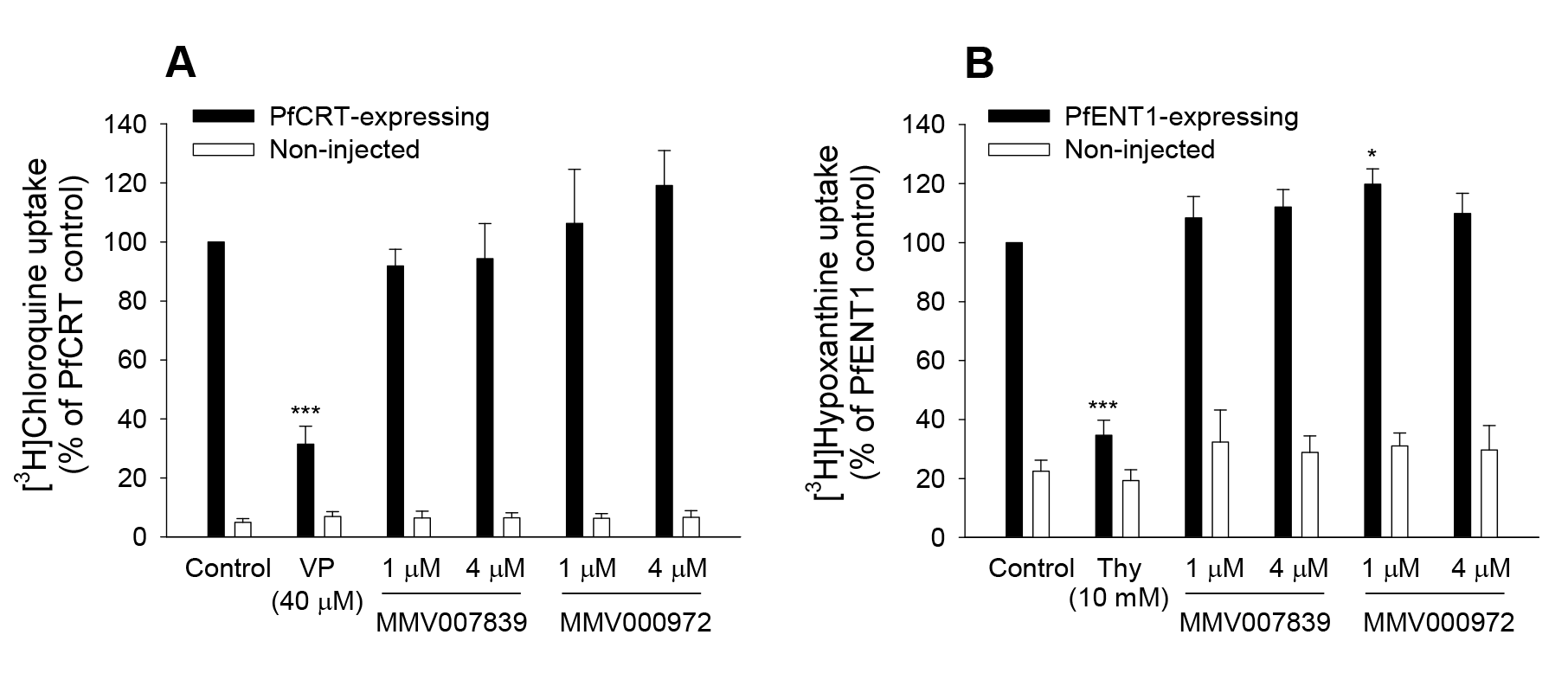

Supplement: S5 Fig — MMV007839 and MMV000972 were tested (both at 1 μM and 4 μM) for their effects on (A) the uptake of [3H]chloroquine by non-injected oocytes (white bars) and by oocytes expressing PfCRT (from the chloroquine-resistant Dd2 strain; black bars) and (B) the uptake of [3H]hypoxanthine by non-injected oocytes (white bars) and by oocytes expressing PfENT1 (black bars). In A, the uptake of [3H]chloroquine (0.25 μM) was measured over 1.5–2 h in the presence of 15 μM unlabelled chloroquine at pH 5.5. Verapamil (VP; an inhibitor of [3H]chloroquine transport by the Dd2 isoform of PfCRT [26] and added here at a concentration of 40 μM) was included as a control. The data are averaged from four to five independent experiments (performed using oocytes from different frogs) and are shown + SEM. In B, the uptake of [3H]hypoxanthine (0.36 μM) was measured over 30 min at pH 6.0. Thymidine (Thy; shown previously to be a substrate for, and hence a competitive inhibitor of, PfENT1 [54], and added here at a concentration of 10 mM) was included as a control. The data are averaged from three to six independent experiments (performed using oocytes from different frogs) and are shown + SEM. Data for each condition were tested for statistical significance compared to the relevant (PfCRT-expressing or PfENT1-expressing) control using one-way ANOVAs with post hoc Tukey tests; *P < 0.05, ***P < 0.001. No significant differences were observed in uptake (within the [3H]chloroquine or the [3H]hypoxanthine datasets) between the non-injected oocyte treatments (one-way ANOVAs with post hoc Tukey tests). (TIF) [file ppat.1006180.s007.tif]

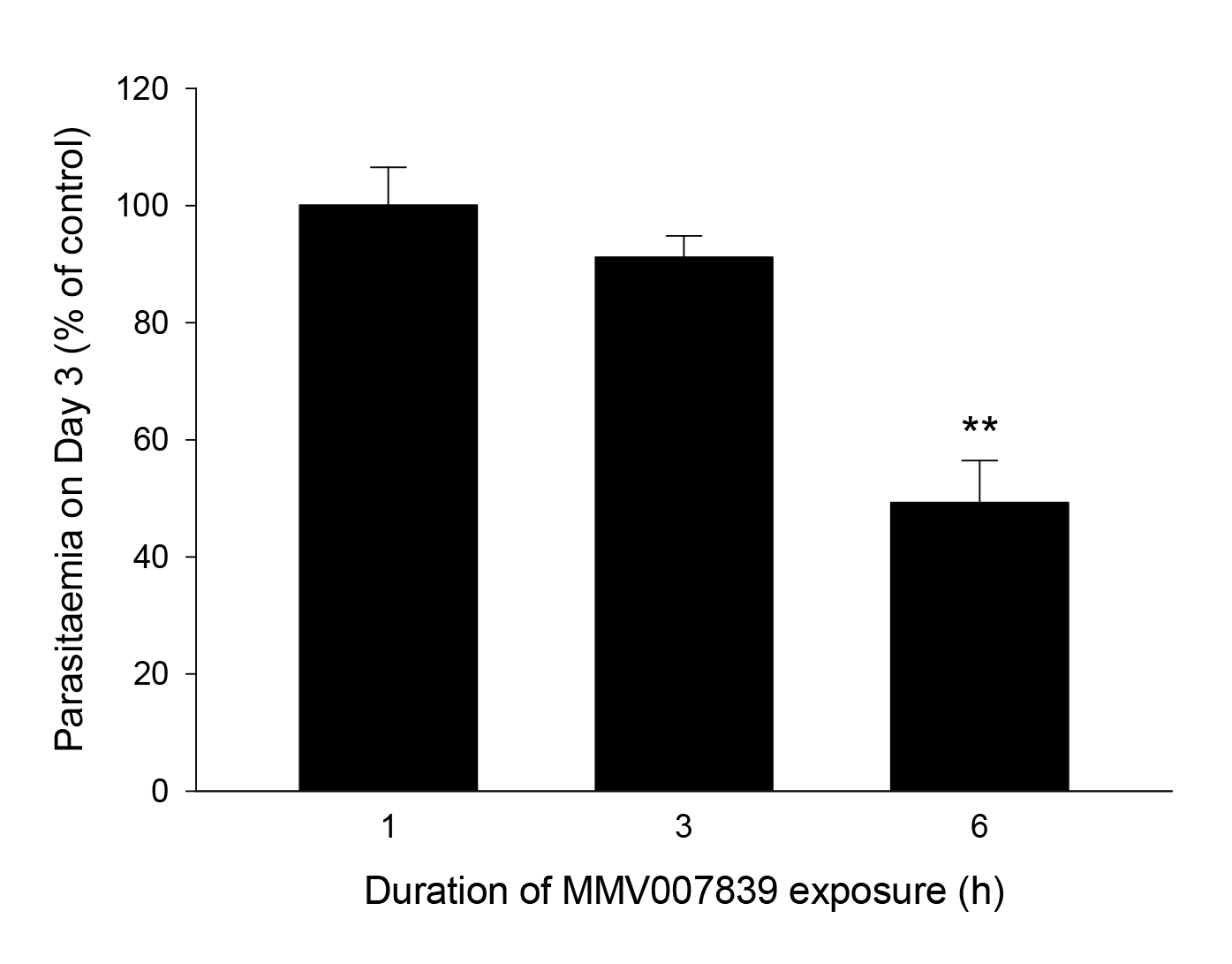

Supplement: S6 Fig — On Day 0, cultures containing erythrocytes infected with 3D7 trophozoites (as well as uninfected erythrocytes) were exposed to either MMV007839 (6 μM) or DMSO (0.02% v/v; solvent control) for 1 h, 3 h or 6 h. The compound (or DMSO) was then removed by washing the cells three times in culture medium and the cells were diluted with fresh culture medium and supplemented with uninfected erythrocytes. The parasitaemias of the cultures were determined on Day 3. Within each experiment, the parasitaemia obtained for the MMV007839-treated culture was normalised to that of the culture treated with 0.02% v/v DMSO for the same length of time. The data are averaged from three independent experiments and are shown + SEM. The (pre-normalised) data for MMV007839- and DMSO-treated cultures were compared at each time point using paired t-tests; **P < 0.01. (TIF) [file ppat.1006180.s008.tif]
